# Transcriptional profiling of lung cell populations in idiopathic pulmonary arterial hypertension

**DOI:** 10.1177/2045894020908782

**Published:** 2020-02-28

**Authors:** Didem Saygin, Tracy Tabib, Humberto E.T. Bittar, Eleanor Valenzi, John Sembrat, Stephen Y. Chan, Mauricio Rojas, Robert Lafyatis

**Affiliations:** 1 Department of Medicine, University of Pittsburgh Medical Center, Pittsburgh, PA, USA; 2 Division of Rheumatology, Department of Medicine, University of Pittsburgh Medical Center, Pittsburgh, PA, USA; 3 Department of Pathology, University of Pittsburgh Medical Center, Pittsburgh, PA, USA; 4 Division of Cardiology, Department of Medicine, University of Pittsburgh Medical Center, Pittsburgh, PA, USA

**Keywords:** pulmonary arterial hypertension, single cell RNA-sequencing, endothelial cells, pericytes

## Abstract

Despite recent improvements in management of idiopathic pulmonary arterial hypertension, mortality remains high. Understanding the alterations in the transcriptome–phenotype of the key lung cells involved could provide insight into the drivers of pathogenesis. In this study, we examined differential gene expression of cell types implicated in idiopathic pulmonary arterial hypertension from lung explants of patients with idiopathic pulmonary arterial hypertension compared to control lungs. After tissue digestion, we analyzed all cells from three idiopathic pulmonary arterial hypertension and six control lungs using droplet-based single cell RNA-sequencing. After dimensional reduction by t-stochastic neighbor embedding, we compared the transcriptomes of endothelial cells, pericyte/smooth muscle cells, fibroblasts, and macrophage clusters, examining differential gene expression and pathways implicated by analysis of Gene Ontology Enrichment. We found that endothelial cells and pericyte/smooth muscle cells had the most differentially expressed gene profile compared to other cell types. Top differentially upregulated genes in endothelial cells included novel genes: *ROBO4, APCDD1, NDST1, MMRN2, NOTCH4*, and *DOCK6*, as well as previously reported genes: *ENG, ORAI2, TFDP1, KDR, AMOTL2, PDGFB, FGFR1, EDN1*, and *NOTCH1*. Several transcription factors were also found to be upregulated in idiopathic pulmonary arterial hypertension endothelial cells including *SOX18, STRA13, LYL1*, and *ELK*, which have known roles in regulating endothelial cell phenotype. In particular, *SOX18* was implicated through bioinformatics analyses in regulating the idiopathic pulmonary arterial hypertension endothelial cell transcriptome. Furthermore, idiopathic pulmonary arterial hypertension endothelial cells upregulated expression of *FAM60A* and *HDAC7*, potentially affecting epigenetic changes in idiopathic pulmonary arterial hypertension endothelial cells. Pericyte/smooth muscle cells expressed genes implicated in regulation of cellular apoptosis and extracellular matrix organization, and several ligands for genes showing increased expression in endothelial cells. In conclusion, our study represents the first detailed look at the transcriptomic landscape across idiopathic pulmonary arterial hypertension lung cells and provides robust insight into alterations that occur in vivo in idiopathic pulmonary arterial hypertension lungs.

## Introduction

Idiopathic pulmonary arterial hypertension (IPAH) is a disease characterized by excessive pulmonary vasoconstriction and pathologic remodeling of small pulmonary arterioles. These changes, in turn, lead to increased intravascular pressures in lung and right ventricular dysfunction. Advances in understanding of IPAH pathogenesis in the last 25 years has led to development of targeted therapies and associated improvement in survival rates in IPAH.^1^ These current targeted treatments include prostacyclin analogs that increase deficient prostacyclin, endothelin receptor antagonists that inhibit endothelin pathway and phosphodiesterase-5 inhibitors, as well as soluble guanylyl cyclase agonists that augment nitric oxide signaling. However, despite recent improvements in management, IPAH remains a devastating disease.^2^ Therefore, understanding IPAH pathogenesis is critical to facilitate development of novel approaches to therapy in the near future.

The pulmonary vascular wall consists of a single layer of endothelial cells (ECs) in the innermost layer, surrounded by pericytes and vascular smooth muscle cells, and extracellular matrix and fibroblasts in the adventitial layer. Pathogenesis of IPAH involves complex interaction between these different cell types. Reduced vessel diameter and increased vascular stiffness are hallmark features, and result from inflammation, proliferation, contraction, thrombosis, and pathological vascular remodeling of pulmonary vessels. EC dysfunction is regarded to play a major role and is characterized by imbalance between production of vasoconstrictors (thromboxane, endothelin, serotonin) versus vasodilators (nitric oxide, prostacyclin, vasoactive intestinal peptide), promoting (fibroblast growth factor-2) versus inhibiting vascular smooth muscle cell proliferation and recruitment of inflammatory cells versus anti-inflammatory effects.^3,4^ Vascular smooth muscle cell proliferation and contraction occurs as a result of activation of hypoxia-inducible factor-1 alpha (HIF1α), downregulation of potassium channels, and upregulation of transient receptor potential channels and anti-apoptotic proteins.^5^ Roles of ECs and vascular smooth muscle cells in IPAH pathogenesis are relatively well-recognized compared to other cell types such as pericytes, fibroblasts, dendritic cells, and lymphoid cells. However, in addition to the defined roles of individual cell types, there is also growing evidence that ECs and pericytes acquire mesenchymal phenotypes similar to vascular smooth muscle cells and play further propagating role in vascular remodeling.^6^

Given dynamic changes in the roles and type of the cells, understanding the transcriptome–phenotype of each lung cell type could shed light on IPAH pathobiology. Previous methodologies used for gene expression analyses are limited by their inability to examine discrete cell types in complex tissues. Single-cell RNA sequencing (scRNA-seq) is a state of art technique that enables analysis of the transcriptomic-phenotype of each of the cell types simultaneously in a tissue. In this study, we sought to define differential gene expression of lung cells in IPAH compared to controls, using scRNA-seq technology.

## Methods

### Study participants

The University of Pittsburgh Medical Center Institutional Review Board (Pittsburgh, PA, USA) reviewed and approved the conduct of this study. Human sample procurement was consistent with the Declaration of Helsinki. Normal and IPAH lungs were obtained under a protocol approved by the University of Pittsburgh. Normal lungs were obtained following rejection as candidate donors for transplant, while IPAH lungs were obtained following removal of recipients' lungs during transplantation surgery. Pulmonary hypertension was defined hemodynamically as mean pulmonary arterial pressure ≥25 mmHg, pulmonary arterial wedge pressure ≤15 mmHg, and pulmonary vascular resistance >3 Woods unit, meeting the World Health Organization criteria for both 2013 and 2018.^7,8^ Furthermore, all patients fulfilled the clinical diagnostic criteria for IPAH, based on objective third-party clinician assessment. Hematoxylin and eosin (H&E) slides of all the lung samples were examined by a pulmonary pathologist for verification of normal lungs and IPAH diagnosis. Clinical information of participants was obtained through electronic medical chart review.

### Processing of lung samples, preparation of single cell libraries, sequencing, and data analysis

Lung samples were brought in Perfadex and processed within 30 min of explant as described previously.^9^ Resulting cell suspensions were loaded into 10 × Genomics Chromium instrument (Pleasanton, CA) for library preparation as described previously.^9^ V1 and V2 single cell chemistries were used per manufacturer's protocol. Libraries were sequenced using the Illumina NextSeq-500 platform. Data analysis was performed using R (version 3.2.1). Seurat, an R package developed for single cell analysis, was used for data analysis, normalization of gene expression, and identification and visualization of cell populations.^10^ Single Cell Regulatory Network Inference (SCENIC), an R package, was used for identification of transcription factors (TFs) regulating transcriptomes.^11^ Cell populations were identified based on gene markers and visualized with t-distributed stochastic neighbor embedding plots. Pathway analysis was performed with Gene Ontology Enrichment Analysis.

## Results

**Fig. 1.**
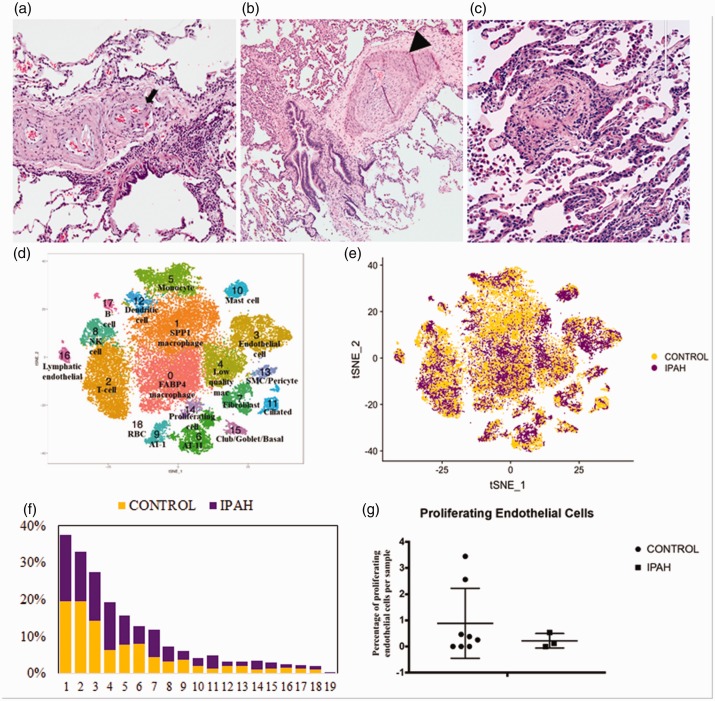
Histopathology of lungs with idiopathic pulmonary arterial hypertension. Histopathology lung explant tissue adjacent to that used for scRNA-seq, showing intimal hyperplasia (arrowhead) and plexiform lesion (arrow) (a–c; magnification 40×, 40 × and 100×, respectively), t-SNE plots showing clusters (d) and origin of cells from control (yellow) and IPAH (purple) lungs (e), proportion of control (yellow) and IPAH (purple) cells in each cluster (f), percentage of proliferating endothelial cells in each control and IPAH lungs (g). IPAH: idiopathic pulmonary arterial hypertension.

Lung samples were obtained from three patients with IPAH and six normal controls (Table 1). Mean pulmonary arterial pressure of the IPAH patients was 61 mmHg (range 56–69) with mean pulmonary capillary wedge pressure of 14 (range 11–19) on right heart catheterization. Mean pulmonary arterial systolic pressures on transthoracic ECHO of these patients was 117 mmHg (range 86–140) associated with severely decreased right ventricular function in all patients, and moderate (two patients) and severe (one patient) tricuspid regurgitation. All IPAH patients were on a phosphodiesterase-5 inhibitor (tadalafil in two and sildenafil in one patient) and a prostacyclin analog (treprostinil), and two patients were also on an endothelin antagonist (bosentan or ambrisentan). H&E-stained slides of each lung sample were examined by the pulmonary pathologist confirming the diagnosis of pulmonary arterial hypertension (PAH) (Fig. 1, panels a–c). We examined on an average 3688 cells per sample from nine subjects. The mean age was 47.5 for controls and 35.6 for IPAH. Both controls and IPAH lungs were processed similarly.

**Table 1.** Characteristics of the lung samples included in the study and right heart catheterization hemodynamic parameters of IPAH patients.

ID	Sample status	Age	Gender	# of cells	Chemistry
1	Normal control	76	Male	884	V1
2	Normal control	56	Male	1196/1314	V1
3	Normal control	55	Male	3327	V2
4	Normal control	57	Female	4481	V2
5	Normal control	18	Male	3383	V2
6	Normal control	23	Female	4516/6071	V2
Total		47.5	4 M, 2 F	3146 cells	2 V1, 4 V2
7	IPAH	21	Male	2988	V1
8	IPAH	50	Female	5398	V2
9	IPAH	36	Female	4307	V2
Total		35.6	1 M, 2 F	4231 cells	1 V1, 2 V2
Hemodynamic parameters of IPAH patients					
Mean right atrial pressure (±SD)					12 ± 4.3
Mean pulmonary arterial pressure (±SD)					61.3 ± 6.8
Mean pulmonary capillary wedge pressure (±SD)					14 ± 4.3

IPAH: idiopathic pulmonary arterial hypertension.

Eighteen distinct clusters of cells were identified, and each cluster contained cells from both control and IPAH lungs (Fig. 1, panels d–f). Each cluster was identified based on presence of known markers as described (Table S1).^9,12,13^ The EC cluster (cluster #3) was identified by strong, distinctive, and overlapping expression of von Willebrand factor (VWF) and cadherin and platelet EC adhesion molecule (PECAM). The pericytes/smooth muscle (pericyte/SMC) cluster (cluster #13) was identified based on expression of *RGS5* and *DES*. Cells in the pericyte/SMC cluster also expressed previously reported pericyte markers: *CCN1, TPM1, TPM2, CALD1*, and *ACTA2* (Table S1).^12,14^ The fibroblast cluster was identified on the basis of *PDGFRA, COL1A*, and *COL1A2* expression (cluster #7).^12,13^ However, we see very little *POSTN* expression in the fibroblast cluster (cluster 7) in IPAH, indicating that these are not an emergent population as seen in systemic sclerosis associated interstitial lung disease^13^ and idiopathic pulmonary fibrosis (IPF, unpublished observations). The monocyte-macrophages and SPP1 macrophage clusters were identified on the basis of expression of *AIF1* and *CD163*, as described.^9^ Proliferating cells, identified as described,^9^ included macrophages and ECs. The IPAH lung samples did not show a significant difference in number of proliferating ECs compared to control lungs (Fig. 1g).

### Differentially expressed genes and pathway analysis in IPAH vs control pulmonary ECs

We compared gene expression in ECs from IPAH to normal control EC gene expression. Because of the limited number of patients undergoing transplantation for IPAH and thus limited number of IPAH samples, and the large scRNA-seq datasets, we developed three algorithms to parse the data prior to statistical analysis. Namely, we selected the most differentially expressed genes between IPAH and control ECs based on: (1) fold difference of expression between IPAH and control pulmonary ECs; (2) absolute expression level; and (3) specificity of the gene for expression by ECs more highly than all other cell types.

Before selection, there were 33,694 genes in the scRNA-seq dataset. For all three algorithms, we filtered out the genes with fold change <1.5, comparing average IPAH to average control expression, and absolute gene expression <0.3, yielding 1980 genes. We then examined differential gene expression using three different approaches (Fig. S1). In the first approach, we selected only genes with a stringent false discovery rate (FDR) of 10% yielding 33 differentially expressed genes (Table 2). These included genes such as *APLN, ENG*, and *KDR* associated with EC growth and angiogenesis, but these genes failed to identify statistically significant pathways on Gene Ontology Enrichment Analysis.

**Table 2.** Top differentially upregulated genes in endothelial cells and Gene Ontology pathways in IPAH.

Endothelial cell genes (10% FDR)		Genes in blood vessel development pathway		Genes in cardiovascular development pathway	
Genes	Fold change	Genes	Fold change	Genes	Fold change
***APLN***	4.94	***PDGFB***	6.23	***PDGFB***	6.23
*C1orf54*	4.04	***APLN***	4.94	***APLN***	4.94
*APCDD1*	3.61	***EDN1***	4.10	***EDN1***	4.10
*ORAI2*	3.41	*COL4A2*	3.67	*SOX18*	4.07
*ENG*	3.29	*LAMA4*	3.46	***ENG***	3.29
*COL6A3*	3.15	***ENG***	3.29	*COL4A1*	3.06
*LAGE3*	2.65	*AMOTL2*	2.89	***NOTCH4***	2.87
*RSBN1L*	2.41	***NOTCH4***	2.87	*SMAD6*	2.79
*KLF16*	2.39	***HDAC7***	2.62	***HDAC7***	2.62
*KNOP1*	2.38	*NDST1*	2.34	*ARHGEF15*	2.11
*GAS6*	2.27	*TNFSF12*	2.21	*PLXND1*	2.09
*LARP1*	2.22	***PRCP***	2.00	*ESM1*	2.06
*FAM60A*	2.21	*KDR*	1.95	***PRCP***	2.00
*PNPLA4*	2.21	***ROBO4***	1.91	***ROBO4***	1.91
*DIMT1*	2.20	***MMRN2***	1.83	*HEG1*	1.88
*GK5*	2.18	***RAMP2***	1.79	*ROM1*	1.88
*LDOC1*	2.14	*FGFR1*	1.79	***MMRN2***	1.83
*TFDP1*	2.10	*LYL1*	1.76	***RAMP2***	1.79
*STRA13*	2.01	*ELK3*	1.76	*ADAM15*	1.74
*UTP4*	1.98	***ARID1A***	1.75	*NOTCH1*	1.53
*TAOK2*	1.96	*PRKACA*	1.55	***BMPR2***	1.52
*KDR*	1.95	***BMPR2***	1.52	*RASIP1*	1.51
*PRRC2A*	1.87	*MYO1C*	1.51		
*DOCK6*	1.84	*SEC24B*	1.50		
*REV1*	1.83				
*FAM89A*	1.83				
*SLC10A3*	1.82				
*WDR77*	1.76				
*IGFBP4*	1.75				
***ARID1A***	1.75				
*SH3KBP1*	1.71				
*VOPP1*	1.51				
*ARL6IP4*	1.50				

FDR: false discovery rate.

Note: Bolded genes are common in multiple pathways and underlined genes are transcription factors and epigenetic modifiers.

In the second approach (algorithm 2), we used a lenient FDR of 60% to improve the identification of genes that might not be identified otherwise due to type II statistical errors, yet still be important. In the third approach (algorithm 3), we analyzed only genes that were more highly expressed in ECs than any other cell type in our scRNA-seq dataset to focus the analysis on these genes expressed the highest in ECs. In this approach, we then applied an even less stringent 45% FDR (Fig. S1). With these latter two approaches, 267 and 107 genes were obtained, respectively, for inputting into pathway analyses.

#### Gene Ontology Enrichment Analysis

We used Gene Ontology Enrichment Analysis to identify relationship between differentially regulated genes in IPAH ECs, inputting the gene lists generated by the different algorithms. Despite the lack of any selection for genes most highly expressed by the EC compartment in algorithm 2, both algorithms selected the same top four differentially regulated pathways in IPAH ECs, all associated with cardiovascular/vascular system development: Cardiovascular System Development, Vasculature Development, Blood Vessel Development, and Blood Vessel Morphogenesis (Table 3). Genes upregulated in ECs from IPAH implicated in both blood vessel development and cardiovascular development included 11 genes: *PRCP, RAMP2, NOTCH4, ROBO4, MMRN2, HDAC7, PDGFB, BMPR2, APLN, ENG*, and *END1* (Table 2). There were also unique genes associated with each of these closely related pathways (13 and 11 genes, respectively, for Blood Vessel and Cardiovascular Development pathways).

**Table 3.** Top upregulated Gene Ontology pathways in endothelial cells in IPAH.

	No. of genes	Fold-change	FDR
Endothelial cell pathways (Algorithm 2)
Blood vessel development	24	3.9	4.89E-04
Cardiovascular system development	24	3.65	5.26E-04
Vasculature development	24	3.72	5.59E-04
Blood vessel morphogenesis	19	3.71	7.02E-03
Tube morphogenesis	23	2.81	3.97E-02
Angiogenesis	15	3.76	5.09E-02
Tube development	26	2.45	1.11E-01
Circulatory system development	26	2.41	1.14E-01
Renal system process	8	5.79	1.80E-01
Positive regulation of epithelial cell migration	9	5.11	1.86E-01
Regulation of epithelial cell migration	11	4.06	1.90E-01
RNA processing	25	2.28	1.91E-01
Extracellular structure organization	15	3.11	1.99E-01
Regulation of endothelial cell migration	9	4.64	2.21E-01
Regulation of blood vessel endothelial cell migration	7	6.21	2.31E-01
Positive regulation of endothelial cell proliferation	7	6.01	2.45E-01
Regulation of endothelial cell proliferation	8	5.05	2.56E-01
Negative regulation of glial cell apoptotic process	3	29.6	2.61E-01
Positive regulation of endothelial cell migration	7	5.7	2.79E-01
Regulation of glial cell apoptotic process	3	26.31	2.95E-01
tRNA processing	8	4.78	3.08E-01
Extracellular matrix organization	13	3.06	3.39E-01
ncRNA processing	14	2.88	3.61E-01
Anatomical structure morphogenesis	45	1.69	4.05E-01
Regulation of cell motility	24	2.13	4.06E-01
Regulation of cell migration	23	2.2	4.12E-01
Cellular process	208	1.14	4.87E-01
RNA metabolic process	37	1.76	4.95E-01
Positive regulation of blood vessel endothelial cell migration	5	7.18	5.02E-01
Endothelial cell pathways (Algorithm 3)
Cardiovascular system development	22	8.64	1.13E-10
Vasculature development	22	8.81	1.55E-10
Blood vessel development	20	8.39	2.35E-09
Blood vessel morphogenesis	18	9.08	8.37E-09
Circulatory system development	24	5.74	1.26E-08
Anatomical structure morphogenesis	36	3.49	2.56E-08
Angiogenesis	15	9.71	1.39E-07
Tube morphogenesis	20	6.32	1.42E-07
Tube development	22	5.36	2.33E-07
Anatomical structure formation involved in morphogenesis	22	5.1	5.18E-07
Regulation of cell motility	21	4.82	3.13E-06
System development	49	2.29	3.26E-06
Regulation of cell migration	20	4.93	5.09E-06
Regulation of locomotion	21	4.45	1.08E-05
Regulation of cellular component movement	21	4.42	1.12E-05
Developmental process	55	1.97	1.92E-05
Anatomical structure development	53	2.02	1.98E-05
Multicellular organism development	50	2.05	3.54E-05
Cellular developmental process	42	2.29	3.87E-05

FDR: false discovery rate.

### Differentially expressed genes and pathway analysis in IPAH vs control pulmonary pericytes

We identified differentially expressed genes between IPAH and control pulmonary pericyte/SMCs, using a similar approach as for ECs, except restricting the analysis to algorithms 1 and 3, because more genes (*n* = 606) were identified using algorithm 2. Selecting genes showing differential expression in IPAH pericytes/SMC compared to control yielded 61 genes using an FDR of 10% (Table 4). Gene Ontology Enrichment Analysis did not yield any significant pathways.

**Table 4.** Top differentially upregulated genes in pericyte/SMCs (10% FDR) and in select Gene Ontology pathways in IPAH.

10% FDR				Circulatory system development		Negative regulation of cell development, differentiation, and developmental process^a^		Extracellular matrix and structure organization^b^	
Genes	Fold change	Genes	Fold change	Genes	Fold change	Genes	Fold change	Genes	Fold change
*ABCA8*	5.44	***MFAP2***	6.06	***ABL1***	1.54	***ABL1***	1.54	*CCDC80*	4.05
*ADGRF5*	2.28	*MRPL23*	2.2	***COL18A1***	3.77	*ACTN4*	2.39	*COL14A1*	1.76
*AFAP1*	3.35	*NEURL1B*	2.45	***COL5A1***	4.82	*CHRD*	3.4	***COL18A1***	3.77
*ANXA6*	1.84	*NFATC3*	2.6	***ECM1***	3.17	***COL5A1***	4.82	***COL5A1***	4.82
*AP3S1*	1.92	*NME3*	1.59	*EDNRA*	2.02	***COL5A2***	2.05	***COL5A2***	2.05
*BCL2L1*	1.54	*NPDC1*	2.64	*ELN*	1.65	***ECM1***	3.17	*EGFL6*	2.3
*C11orf74*	3.37	*NSMCE4A*	2.31	*FBXW7*	3.43	*FBXW7*	3.43	***ELN***	1.65
*CAND1*	2.07	*ORAI2*	4.02	*FHL2*	1.64	*FKBP4*	1.65	*ITGA1*	1.94
*CBWD2*	3.27	***PBX1***	6.93	*JAG1*	5.31	*FRZB*	3.83	*ITGA8*	3.19
*CDC5L*	2.7	*PFDN6*	3.63	*LTBP1*	1.77	*GDF10*	1.53	*ITGB5*	1.82
*CHTF8*	1.62	*PGM5*	2.42	*MCAM*	1.57	*GDI1*	1.96	*MFAP2*	6.06
***COL18A1***	3.77	*PHF14*	3.77	*MED1*	7.02	*HOOK3*	1.51	*MFAP4*	3.2
*CREBZF*	2.83	*PQLC1*	6.9	*MEIS1*	6.64	*ITM2C*	2.68	***MYH11***	1.9
*CUL5*	3.18	*PQLC3*	2.81	***MYH11***	1.9	*JAG1*	5.31	***NF1***	3.38
*DCTPP1*	3.39	*PTPRA*	2.76	***NF1***	3.38	***LPAR1***	8.14	*NPNT*	1.77
*DEXI*	4.81	*RBM14*	2.71	*NTRK2*	1.7	*MED1*	7.02	*OLFML2B*	1.86
*DTX3*	1.86	*RERG*	3.08	*NTR3*	1.74	*MEIS1*	6.64	*PLTP*	3.3
*EFEMP1*	2.99	*RFC2*	2.54	*PCSK5*	6.71	***NF1***	3.38	***PTK2***	3.52
*FLII*	3.85	*RFK*	2.34	*PDGFRB*	1.76	*NTRK3*	1.74	***SPARC***	1.59
*FUNDC1*	2.02	*RPL26L1*	2.2	*PGF*	2.44	*PBX1*	6.93		
*GTPBP6*	1.93	*RSRC1*	3.54	*PLXDC1*	3.06	***PTK2***	3.52		
*HGSNAT*	5.2	*SAFB2*	2.29	***PTK2***	3.52	*RUNX1T1*	2.89		
*HOTAIRM*	1.59	*SDHAF1*	2.37	*SLIT3*	2.26	***SMAD4***	3.01		
***ITGA1***	1.94	*SLC30A5*	1.94	***SMAD4***	3.01	*SNAPIN*	8.99		
*KAT5*	1.65	*SLC41A3*	2.97	*SMARCD3*	5.41	***SPARC***	1.59		
*KLHDC10*	2.97	*SMC6*	2.37	***SPARC***	1.59	*STARD13*	5.89		
***LPAR1***	8.14	*SNAPC3*	1.9	***TBX2***	1.62	***TBX2***	1.62		
*LTBP3*	2.21	*SNX21*	5.12	***THY1***	7.72	***THY1***	7.72		
*MAGED1*	4.62	*TCF7L2*	2.31	***ZFP36L1***	1.66	***ZFP36L1***	1.66		
*METRN*	6.07	*TMEM67*	16.72						
		*WRB*	2.53						

a *ABL1, ECM1, SPARC, STARD13*, and *TBX2* were found only in negative regulation of developmental process pathway.

b *PLTP* gene was found only in extracellular structure organization pathway.

Note: Bolded genes are common in multiple pathways.

FDR: false discovery rate.

Filtering 33,694 genes with fold change >1.5, absolute gene expression >0.3, and selecting only genes that were expressed most highly in pericyte/SMCs with 45% FDR yielded 206 genes (Fig. S1, algorithm 3). Inputting these genes into Gene Ontology Enrichment Analysis yielded top differentially regulated pathways, including Negative Regulation of Cell Development, Developmental Processes and Cell Differentiation, Anatomical Structure Morphogenesis, Extracellular Matrix Organization, Extracellular Structure Organization, and Circulatory System Development (Table S2); 29 genes were found to be in Circulatory System Pathway (Table 4). Many of the genes in this pathway overlapped with those seen in the other pathways (Table 4, bolded genes).

### Differentially expressed genes and pathway analysis in IPAH vs control pulmonary fibroblasts

We identified differentially expressed genes in IPAH compared to control lung fibroblasts, since adventitial fibroblasts have been implicated in PAH pathogenesis.^15^ Using the same approach as that for analyzing pericyte/SMCs, genes were identified showing higher expression in IPAH compared to control fibroblasts, using an FDR of 10%, and genes most highly expressed by fibroblasts comparing IPAH with control fibroblasts using an FDR of 45% (Fig. S1, Table S3), the former not yielding significant pathways on Gene Ontology Enrichment. Gene Ontology Enrichment Analysis inputting upregulated fibroblast genes at FDR of 45% identified several pathways, including Gene Matrix Organization, as well as Regulation of WNT and Response to TGFβ (Table S4A). Ten genes were associated with the extracellular matrix pathway and three and five genes associated with the Regulation of WNT Signaling and Response to TGFβ Signaling pathways, respectively (Table S4B).

### Differentially expressed genes and pathway analysis in IPAH vs control pulmonary monocyte-macrophages and SPP1 macrophages

We sought to identify differentially expressed genes between IPAH and control pulmonary monocyte-macrophage, and SPP1 macrophages based on our previous observations of these macrophage subpopulations,^9^ using a similar approach to above. Filtering 33,694 genes with fold change >1.5, absolute gene expression >0.3, and selecting genes that were differentially expressed at a 10% FDR threshold most highly in monocyte-macrophages yielded 16 genes and for SPP1 macrophages, 18 genes (Table S5). Selecting genes more highly expressed in each of the SPP1 and monocyte-macrophage subsets compared to other cell cluster yielded only 134 and 17 genes, respectively, even without an FDR selection. As none of these selection criteria yielded statistically significant pathways on Gene Ontology analysis, we did not pursue further analyses.

### Predicted TFs in regulating IPAH transcriptome

**Fig. 2.**
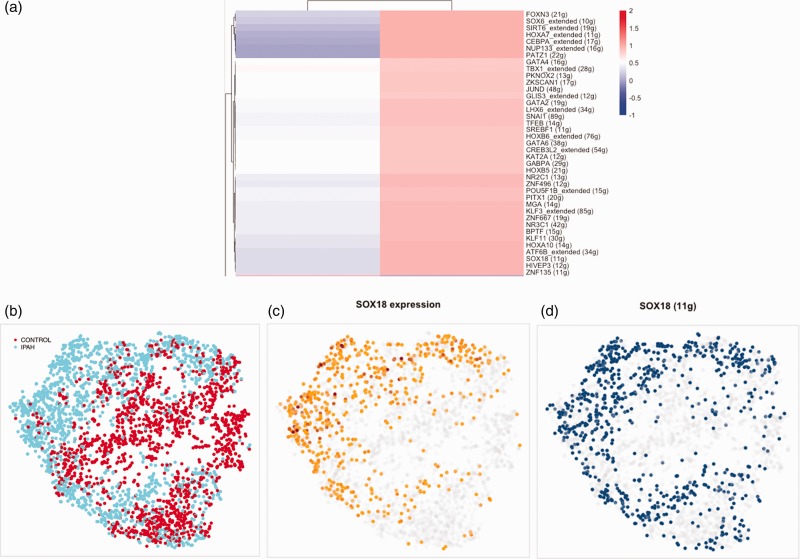
Regulon and predicted transcription factors regulating endothelial cell transcriptome expression in IPAH endothelial cells. Cluster of transcription factors predicted to be upregulated in IPAH endothelial cells (panel a). T-SNE clustering by regulon (panels b–d), showing the origin of the cells: IPAH (blue) versus control (red) lung explants (panel b). SOX18 expression in regulon t-SNE is indicated by intensity of yellow/brown color (panel c). Level of SOX18 regulon activity in SOX18 regulon t-SNE is indicated by intensity of blue color (panel d). IPAH: idiopathic pulmonary arterial hypertension.

In order to identify TFs that might play important roles in the dysregulated IPAH EC phenotype, we analyzed the EC transcriptome data using SCENIC, a computational method for gene regulatory network reconstruction from scRNA-seq datasets.^11^ SCENIC implicated 39 TFs as regulating IPAH EC transcriptome (Fig. 2a). However, of the TFs upregulated in IPAH ECs analyzed by SCENIC, including *SOX18, STRA13, LYL1, ELK*, and *TFDP1*, only *SOX18* showed a pattern of regulon expression that overlaid strongly with IPAH ECs (Fig. 2b). This mirrored the level of expression of *SOX18* in IPAH cells (Fig. 2c and d).

### Genes associated with hereditary PAH are selectively expressed in ECs, pericytes/SMC, and dendritic cells

**Fig. 3.**
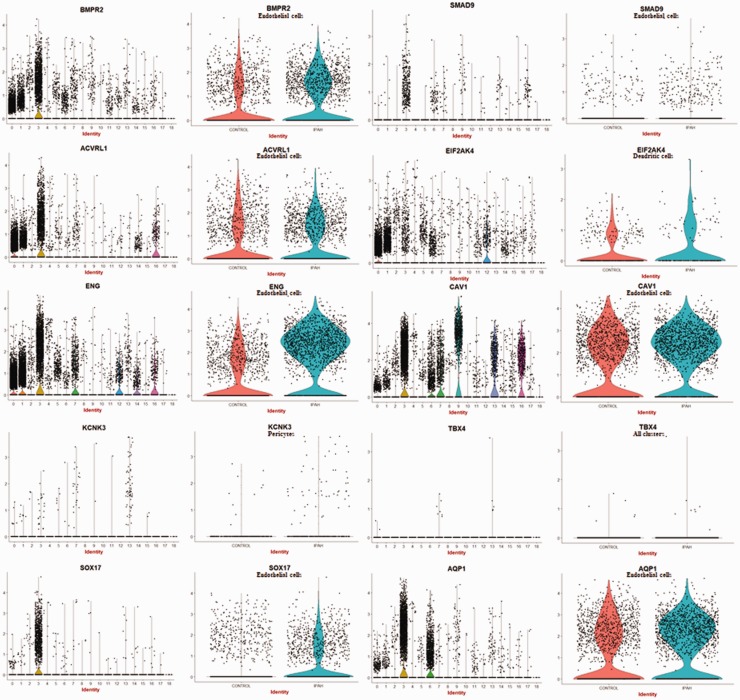
Expression of genes associated with hereditary IPAH in different cell types. Violin plots indicate expression of genes associated with hereditary IPAH in each cluster (cluster numbers as in Fig. 1) of cells from both IPAH and control lungs (panels on left). Violin plots showing expression of the cell type with the highest expression in left panels, show expression divided between control and IPAH lungs (panels on the right). For most genes, highest expression is cluster #3 (endothelial cells). For *KCNK3*, highest expression is in pericytes (cluster #13). For *EIF2AK4* highest expression is in dendritic cells (cluster #12).

To clarify the cell types expressing genes that have been shown mutated in hereditary PAH, we examined their expression in our scRNA-seq data. This analysis supported the highly selective, though not exclusive, expression of *BMPR2, ACVRL1*, and *ENG* on ECs, with modestly increased expression of *BMPR2* (1.52-fold), no change in *ACVRL*1 (1.0-fold), and upregulated expression of *ENG* (3.29-fold) by PAH compared to control ECs (Fig. 3). These three genes were also expressed at lower levels by macrophages. *SMAD9* and *SOX17* were expressed almost exclusively by ECs, and *SMAD9*, but not *SOX17*, upregulated on IPAH ECs (*SMAD9*: 1.99-fold; *SOX17*: 1.05-fold). *AQPN* was also expressed selectively on ECs, but also by alveolar type II cells, showing relatively little difference in expression comparing IPAH to control ECs (1.20-fold). In contrast, *KCNK3* was most highly expressed by pericytes and highly upregulated in IPAH compared to control ECs (8.18-fold). EIFA2K4 was expressed by many cell types, including macrophages and dendritic cells. *CAV1* was expressed highly by ECs but more highly by type I alveolar cells, as well as pericyte/SMCs and lymphatic ECs. *TBX4* was detected only at very low levels in fibroblasts and pericyte/SMCs. Thus, of the genes associated with hereditary PAH, *ENG* and *SMAD9* were the most upregulated in IPAH ECs and *KCNK3* in pericytes, with the other genes showing no or modest regulation.

### ScRNA-seq data are validated by and extend bulk microarray data

The recent analysis of a large database of microarray data from IPAH and control lungs provided a robust opportunity to confirm regulation of genes found in our dataset.^16^ Our scRNA-seq data in turn provided the opportunity to significantly extend bulk lung gene expression observations by showing which cells are expressing genes shown to be regulated in bulk lung expression data. To show the utility in this approach, we compared bulk microarray RNA expression by IPAH (*n* = 31) and failed donor lungs (*n* = 25, NCBI GEO, GSE117261^16^) with our scRNA-seq data, selecting the 10 most statistically significant upregulated genes in bulk microarray for comparison (Table S6). Two of the most statistically significant upregulated genes were Hemoglobin genes (hemoglobin subunit beta and hemoglobin subunit alpha 2///hemoglobin subunit alpha 1), which were both expressed most highly in scRNA-seq erythrocyte cluster #18 (not shown), and one of the genes, *GGTA1P*, was not seen in the scRNA-seq data. Six of the seven other genes showed increased expression in specific cells types: *FZD7* in IPAH pericyte/SMCs, *ECM2*, and *RARRES2* in IPAH fibroblasts, *LTBP1* and *PDE3A* in IPAH fibroblasts and pericyte/SMCs, and *PDE7B* in IPAH ECs, fibroblasts, pericyte/SMCs, and mast cells (Fig. S2). We include a full dataset of gene expression in each cluster, each sample, and each gene (Table S7A) in addition to average expression of each gene in IPAH and control lungs for each gene (Table S7B) providing reference databases for investigators (see http://dom.pitt.edu/rheum/centers-institutes/scleroderma/systemicsclerosiscenter/database/).

## Discussion

Comparing transcriptomic profiles of different cell types in healthy and IPAH lungs by scRNA-seq, we found that ECs and pericyte/SMCs displayed the most differentially expressed genes between IPAH and normal lungs compared to other cell types. As such, our results reinforce the important role of ECs and pericyte/SMCs in IPAH pathogenesis. Among the EC genes that were most differentially upregulated in IPAH: *ENG, ORAI2, TFDP1, KDR, AMOTL2, PDGFB, FGFR1, EDN1*, and *NOTCH1* have been previously reported in the literature and support the broader validity of our results.^17–23^ The Pulmonary Hypertension Breakthrough Initiative has provided the largest transcriptome study data to date.^16^ Our data complement this study, representing the first close look at the transcriptomic landscape across IPAH lung cells in vivo, thus allowing for cell-specific analysis not possible with prior findings in cultured cells or whole lung transcriptomic analysis.

Our data show several cytokine and cytokine receptor genes upregulated in IPAH ECs, likely contributing in an autocrine fashion to the altered phenotype of ECs as well as in a paracrine fashion to pericyte/SMC hyperplasia in IPAH. This includes upregulated EC expression of platelet derived growth factor-beta (PDGF-β), endothelin-1, and Apelin (*APLN*), and of the receptors VEGFR-2 (*KDR*), *BMPR2*, *ENG*, and *FGFR1*. As such, EC expression in IPAH appears to strongly support roles for multiple growth factors that regulate angiogenesis, robustly supported also by the pathway analyses.

*FGFR1* has been shown upregulated in pulmonary arterial ECs^24^ associated with decreased *APLN* expression. In recently reported bulk data,^16^ *FGFR1* showed 1.19-fold and *APLN* showed 1.56-fold increased expression. Our data showed both of these genes increased in IPAH ECs. APLN, a peptide that binds to apelin receptor (APLNR),^25^ promotes angiogenesis through activation of extracellular-signal-regulated kinases, Akt, and p70S6kinase, and has vasodilator properties through induction of nitric oxide release.^26^ Previous studies showed reduced *APLN* mRNA levels and *APLN* expression in cultured ECs from IPAH compared to control lungs.^24^ Furthermore, administration of APLN was shown to reverse PAH in mice. Our study challenges these data on the role of APLN in IPAH, indicating that not only APLN but also the Apelin cleaving enzyme Lysosomal Pro-X carboxypeptidase (PRCP),^27^ a protein also described to promote angiogenesis and vascular repair,^28^ show elevated expression by PAH ECs.

IPAH ECs also showed upregulated expression of endoglin (*ENG*, 3.29-fold), encoding a Transforming growth factor-beta (TGFβ) signaling co-receptor that is highly expressed on ECs and controls EC differentiation, proliferation, and angiogenesis. Loss of function *ENG* mutations leads to hereditary hemorrhagic telangiectasia (HHT) type 1, an autosomal dominant syndrome characterized by vascular dysplasia associated with PAH.^29^ Relatively frequent mutations in the *ENG* gene associated with PAH have been described, although the role of these in disease is uncertain.^18^ However, in PAH, *ENG* expression has been reported as elevated on IPAH ECs, consistent with our scRNA-seq observation.^30^ In distinction from HHT patients in whom deficient *ENG* is associated with PAH, in mice *ENG* deficiency is protective for hypoxic pulmonary hypertension. The apparent paradox between the development of PAH in both settings of deficient and upregulated gene expression has been considered in regard to Fibroblast growth factor (FGF).^31^ A similar paradox is presented by our data, which show increased *ENG*, in PAH, even though genetic data indicate that deficient function of these receptors is associated with PAH. In addition to elevated *ENG* expression, PAH ECs also showed modestly increased expression of *BMPR2* (1.52-fold), the most common gene mutated in familial PAH and associated with deficient function. In comparison, bulk lung IPAH mRNA *BMPR2* expression showed no change (1.00-fold) compared to control lungs (from NCBI GEO, GSE117261^16^). These results suggest an uncoupling between *BMPR2* mRNA and protein expression, as BMPR2 protein expression has been shown previously to be downregulated in IPAH by immunohistochemistry.^32^

In addition to the several cytokines and cytokine receptors already implicated in IPAH, we saw upregulated expression of several other genes involved in regulating EC growth: *ROBO4*, an endothelial receptor that regulates endothelial migration^33^ and stabilizes the endothelium by opposing signaling by *VEGF*;^34^ *APCDD1*, an inhibitor of Wnt signaling pathway in which overexpression is associated with increased paracellular barrier permeability by retinal endothelium and shown to coordinate the timing of vascular pruning and barrier maturation;^35^ *NDST1*, a bifunctional enzyme that catalyzes both the N-deacetylation and the N-sulfation of glucosamine of the glycosaminoglycan in heparan sulfate, which regulates BMP signaling and internalization in lung development by affecting the binding of BMP to extracellular matrix;^36^ and *MMRN2*: (Multimerin2), also known as Endoglyx-1, an extracellular matrix glycoprotein that binds to and inhibits the activity of VEGFA.^37,38^

Two genes implicated directly (*NOTCH4*) or indirectly (*DOCK6*) in the NOTCH pathway were upregulated. *NOTCH4* is expressed exclusively in ECs as opposed to other NOTCH subtypes. Constitutively active endothelial *NOTCH4* inhibits EC apoptosis,^39^ and is associated with brain and lung arteriovenous malformations in mice,^40^ suggesting roles of *NOTCH4* in EC survival and vascular integrity. *DOCK6* is a guanine nucleotide exchange factor regulator of Cdc42 that activates Rho family guanosine triphosphatases, Rac1 and Cdc42, and is required for normal function of the actin cytoskeletal structure. Autosomal recessive forms of Adams–Oliver syndrome can be due to mutations in *EOGT*, which encodes a component of Notch pathway, or *DOCK6* mutations.^41–43^ Autosomal dominant forms are caused by mutations in *NOTCH1, RBPJ*, or *DLL4* (all NOTCH pathway components) or *ARHGAP31*, which encodes another Rho GTPase regulator. Patients with Adams–Oliver syndrome who have *DOCK6* mutations develop dilated surface blood vessels, pulmonary or portal hypertension, and retinal hypervascularization.

Several TFs upregulated in IPAH ECs, including *SOX18, STRA13, LYL1*, and *ELK*, have known roles in regulating EC phenotype. *SOX18*, expressed four times higher in IPAH than control ECs, regulates vasculature development and endothelial barrier integrity.^44–46^ In an ovine model of congenital heart disease with shunt, *SOX18* expression was upregulated in pulmonary arterial ECs, correlating with increased trans-endothelial resistance.^47^ Thus, upregulation of *SOX18* in IPAH might be a primary alteration driving EC microangiopathy and obliterative changes, or an adaptive change to high shear stress. Notably, of all the TFs analyzed by SCENIC, it showed the most robust regulon and associated increased expression in IPAH ECs, suggesting that it plays a critical role in the altered IPAH transcriptome. *STRA1*3 is a basic helix-loop-helix (bHLH) TF that is upregulated during hypoxia by HIF1α/VHL. It plays important roles in the regulation of cell proliferation, differentiation, and apoptosis.^48^ Both Stat1 and Stat3 are targets of *STRA13, STRA13* mediating hypoxic repression of Stat1,^49^ *STRA13* also binding to Stat3.^50^ *LYL1* is a bHLH TF and a major regulator of adult neovascularization.^51^ *LYL1* and *TAL1*, another bHLH TF, along with a cofactor LIM-only-2 protein regulate EC transcription of Angiopoietin-2 a major regulator of angiogenesis.^52^ *ELK3* is a transcriptional repressor that is downregulated during hypoxia, releasing repression of several genes and leading to increased expression of Egr1 and VEGF, as well as PHD2, PHD3, and Siah2 destabilizing HIF1α.^53–55^ Thus, its elevated expression is surprising and might be expected to inhibit angiogenesis. *TFDP1*, a TF that regulates proliferation, apoptosis, and differentiation of myeloid cells was also upregulated in ECs.^56–59^ *TFDP1* complexes with E2F and regulates a series of genes involved in cell cycle, suggesting that its elevation in IPAH might promote EC proliferation.^60^ Most likely, these TFs work in combination to alter the transcriptome and phenotype of IPAH ECs.

In addition to insights in TFs regulated in IPAH, upregulated expression of *FAM60A* and *HDAC7* indicate important epigenetic changes are occurring in IPAH ECs. *FAM60A/SINHCAF* is part of the SIN3A–HDAC complex, a master transcriptional repressor that is required for a complete response to hypoxia.^61^ *FAM60A/SINHCAF* specifically represses HIF2α mRNA and protein expression by interacting with the TF SP1 and recruitment of HDAC1 to the HIF2α promoter.^62^ *HDAC7* is a class IIa histone deacetylase with a well described role in regulating angiogenesis. Mice deleted of *HDAC7* show loss of endothelial cell–cell adhesion, vascular dilation, and rupture.^63^ *HDAC7* is also required in vitro for EC migration and tube formation.^64^ *HDAC7* epigenetically regulates *MMP10* and *AKAP12*, a suppressor of angiogenesis.^65^ VEGFA stimulates HDAC7 phosphorylation and shuttling into the nucleus.^66^

Examining genes showing increased expression by pericyte/SMCs, meeting the 10% FDR revealed several genes implicated in regulating cellular apoptosis: *BCL2L1*,^67^ *FUNDC1*,^68^ and *KLHDC10*;^69^ Lysophosphatidic acid signaling: *LPAR1*;^70^ and muscle and smooth muscle cell differentiation and growth: *MAGED1*^71^ and *NFATC3*.^72^ Several TFs and epigenetic regulators were upregulated in IPAH pericyte/SMCs. *PBX1* encodes a TF that works cooperatively with *KLF4* and *MEIS2*;^73^ cooperates with *MYOD1* in myoblast differentiation;^74^ is expressed widely in developing mesenchymal tissues including smooth muscle; and its deletion leads to pulmonary hypertension associated with elevated *MYH11* and *EDN1* in whole lungs.^75^ *PHF14*, suggested to be an epigenetic regulator through binding to histones, has been implicated in lung development and its deletion in a neonatal case of pulmonary hypertension.^76,77^ *SAFB2* encodes a transcriptional repressor^78^ and *TCF7L2*, a TF involved in Wnt signaling, mutated in diabetes mellitus type 2, and important in pancreatic pericyte function.^79^

Examining genes in IPAH pericytes ECM pathway analysis showed upregulated expression of multiple ECM proteins and integrins, suggesting that these cells have converted to a profibrotic phenotype characterized in particular by increased expression of *COL14A1, COL18A1, COL5A1, COL5A2, ELN, ITGA1, ITGA8, ITGB5, MFAP2, MFAP4, OLFML2B*, and *SPARC*. Notably, COL18A1 is cleaved to form endostatin, an inhibitor of angiogenesis.^80^

IPAH pericytes also expressed several ligands for genes showing increased expression in IPAH ECs. *SLIT3* was upregulated on IPAH pericyte/SMCs while its cognate ligand *ROBO4* was upregulated on IPAH EC;^81^ *PDGFRB* was upregulated in IPAH pericyte/SMCs while its growth factor ligand *PDGFB* was upregulated in IPAH ECs; and *JAG1* was upregulated on IPAH pericyte/SMCs while its cognate ligand *NOTCH1* was upregulated on IPAH ECs. Thus, it appears IPAH pericyte/SMCs might be acting to facilitate or alter the IPAH EC phenotype through several of these interacting receptor–ligand pairs. Finally, both *ITGA8* and its cognate ligand *NPNT*, associated with arrector pili muscle development in skin,^82^ were upregulated on IPAH pericyte/SMCs, suggesting possible autocrine role in pericyte hypertrophy.

Monocyte-derived macrophages have been implicated in PAH in several studies in hypoxia-induced disease,^83–86^ and these cells implicated as potential mesenchymal progenitors.^85^ We did not see evidence for monocyte-macrophage co-expression of *COL1A1* to suggest a population of monocyte-macrophage mesenchymal progenitors. Changes in gene expression in macrophage populations in IPAH lungs were modest and relatively few differentially expressed genes met a 10% FDR, compared to EC and pericyte/SMC regulated genes, suggesting that in late stage IPAH, macrophage populations may not be as active or may display more heterogeneous or individualized roles not discernible through this analysis.

There are several limitations to this study. Most important is the relatively small sample size. As IPAH patients come to transplant relatively infrequently, this is an inherent limitation related to their clinical course. To mitigate this limitation, we placed other limitations on the data, examining only upregulated genes, and for pathway analysis allowing liberal FDRs, which leads to a lack of conclusive statistical rigor for some of the genes associated with the pathway analyses. This is partially mitigated by the highly statistically significant pathway associations of these genes. However, the most reliable alterations in gene expression are represented by our initial analysis using a 10% FDR. The other major limitation is that lung explants represent end-stage disease in patients who are heavily treated with vasodilators. It is possible that some of the changes in gene expression identified in this study are representative of only late-stage disease and/or are changes that are reactive to therapies. For example, it is known that treatment with endothelin antagonists is associated with increased plasma levels of endothelin-1.^87^ As IPAH patients almost never undergo lung biopsies, these limitations cannot be overcome under current standard of care. Despite these limitations, this approach analyzes the cells under conditions in which there is ongoing pulmonary hypertension and assesses gene expression within hours of lung explant. As such, it provides a robust, transcriptomic landscape of IPAH lungs, showing major changes in gene expression of ECs, pericyte/SMCs, and fibroblasts in IPAH pathology, implicating multiple novel genes in IPAH pathogenesis.

## Supplemental Material

PUL908782 Supplemental Material - Supplemental material for Transcriptional profiling of lung cell populations in idiopathic pulmonary arterial hypertension Click here for additional data file. Supplemental material, PUL908782 Supplemental Material for Transcriptional profiling of lung cell populations in idiopathic pulmonary arterial hypertension by Didem Saygin, Tracy Tabib, Humberto E.T. Bittar, Eleanor Valenzi, John Sembrat, Stephen Y. Chan, Mauricio Rojas and Robert Lafyatis in Pulmonary Circulation

PUL908782 Supplemental Material - Supplemental material for Transcriptional profiling of lung cell populations in idiopathic pulmonary arterial hypertension Click here for additional data file. Supplemental material, PUL908782 Supplemental Material for Transcriptional profiling of lung cell populations in idiopathic pulmonary arterial hypertension by Didem Saygin, Tracy Tabib, Humberto E.T. Bittar, Eleanor Valenzi, John Sembrat, Stephen Y. Chan, Mauricio Rojas and Robert Lafyatis in Pulmonary Circulation
