# Supplementary material for: Transcriptional profiling of lung cell populations in idiopathic pulmonary arterial hypertension
Source: Pulm Circ. 2020 Feb 28;10(1):???. doi: 10.1177/2045894020908782 (PMC7052475; doi:10.1177/2045894020908782)
Supplement: PUL908782 Supplemental Material - Supplemental material for Transcriptional profiling of lung cell populations in idiopathic pulmonary arterial hypertension [file PUL908782_Supplemental_Material.pdf]

**Supplementary Table 1. The top 50 most differentially upregulated genes in each cluster**

| | Gene | avg_logFC | Cluster ID | p_val | pct.1 | pct.2 | p_val_adj |
|---|---|---|---|---|---|---|---|
| 1 | *FABP4* | 1.98 | 0 | 0 | 0.94 | 0.29 | 0 |
| 2 | *C1QB* | 1.63 | 0 | 0 | 0.98 | 0.36 | 0 |
| 3 | *C1QA* | 1.47 | 0 | 0 | 0.98 | 0.39 | 0 |
| 4 | *INHBA* | 1.32 | 0 | 0 | 0.78 | 0.08 | 0 |
| 5 | *C1QC* | 1.25 | 0 | 0 | 0.93 | 0.25 | 0 |
| 6 | *CD52* | 1.24 | 0 | 0 | 0.97 | 0.51 | 0 |
| 7 | *APOC1* | 1.23 | 0 | 0 | 0.99 | 0.43 | 0 |
| 8 | *RP11-598F7.3* | 1.17 | 0 | 0 | 0.83 | 0.10 | 0 |
| 9 | *FBP1* | 1.15 | 0 | 0 | 0.94 | 0.31 | 0 |
| 10 | *MSR1* | 1.09 | 0 | 0 | 0.88 | 0.18 | 0 |
| 11 | *MCEMP1* | 1.06 | 0 | 0 | 0.92 | 0.25 | 0 |
| 12 | *RBP4* | 1.06 | 0 | 0 | 0.61 | 0.05 | 0 |
| 13 | *CD74* | 0.97 | 0 | 0 | 0.99 | 0.71 | 0 |
| 14 | *ALOX5AP* | 0.96 | 0 | 0 | 0.96 | 0.44 | 0 |
| 15 | *HLA-DRA* | 0.95 | 0 | 0 | 0.99 | 0.66 | 0 |
| 16 | *SERPINA1* | 0.94 | 0 | 0 | 0.92 | 0.31 | 0 |
| 17 | *CD68* | 0.92 | 0 | 0 | 0.96 | 0.36 | 0 |
| 18 | *TYROBP* | 0.92 | 0 | 0 | 0.99 | 0.54 | 0 |
| 19 | *RND3* | 0.92 | 0 | 0 | 0.52 | 0.07 | 0 |
| 20 | *TREM1* | 0.91 | 0 | 0 | 0.87 | 0.21 | 0 |
| 21 | *HLA-DQB1* | 0.91 | 0 | 0 | 0.95 | 0.36 | 0 |
| 22 | *ACP5* | 0.89 | 0 | 0 | 0.97 | 0.33 | 0 |
| 23 | *MARCO* | 0.89 | 0 | 0 | 0.94 | 0.27 | 0 |
| 24 | *GRN* | 0.89 | 0 | 0 | 0.95 | 0.45 | 0 |
| 25 | *HLA-DRB5* | 0.88 | 0 | 0 | 0.81 | 0.37 | 0 |
| 26 | *PHLDA3* | 0.88 | 0 | 0 | 0.80 | 0.10 | 0 |
| 27 | *FTL* | 0.86 | 0 | 0 | 1.00 | 0.99 | 0 |
| 28 | *HLA-DQA1* | 0.85 | 0 | 0 | 0.90 | 0.25 | 0 |
| 29 | *HLA-DRB1* | 0.85 | 0 | 0 | 0.97 | 0.53 | 0 |
| 30 | *CXCL16* | 0.85 | 0 | 0 | 0.92 | 0.28 | 0 |
| 31 | *SPI1* | 0.84 | 0 | 0 | 0.92 | 0.28 | 0 |
| 32 | *VSIG4* | 0.84 | 0 | 0 | 0.92 | 0.26 | 0 |
| 33 | *MIR3945HG* | 0.84 | 0 | 0 | 0.66 | 0.11 | 0 |
| 34 | *SNX10* | 0.84 | 0 | 0 | 0.92 | 0.25 | 0 |
| 35 | *MS4A7* | 0.82 | 0 | 0 | 0.96 | 0.32 | 0 |
| 36 | *GCHFR* | 0.81 | 0 | 0 | 0.96 | 0.35 | 0 |
| 37 | *FABP3* | 0.81 | 0 | 0 | 0.37 | 0.05 | 0 |
| 38 | *HLA-DPA1* | 0.80 | 0 | 0 | 0.98 | 0.54 | 0 |
| 39 | *LPL* | 0.80 | 0 | 0 | 0.74 | 0.11 | 0 |
| 40 | *CCL18* | 0.80 | 0 | 0 | 0.78 | 0.26 | 0 |
| 41 | *SCD* | 0.78 | 0 | 0 | 0.72 | 0.13 | 0 |
| 42 | *CSTB* | 0.77 | 0 | 0 | 0.99 | 0.64 | 0 |

| | | | | | | | |
|---|---|---|---|---|---|---|---|
| 43 | *ALDH2* | 0.77 | 0 | 0 | 0.92 | 0.36 | 0 |
| 44 | *HLA-DMA* | 0.76 | 0 | 0 | 0.94 | 0.36 | 0 |
| 45 | *CES1* | 0.76 | 0 | 0 | 0.67 | 0.14 | 0 |
| 46 | *CYP27A1* | 0.76 | 0 | 0 | 0.86 | 0.20 | 0 |
| 47 | *LY6E* | 0.75 | 0 | 0 | 0.92 | 0.40 | 0 |
| 48 | *TSPO* | 0.75 | 0 | 0 | 0.97 | 0.58 | 0 |
| 49 | *PCOLCE2* | 0.74 | 0 | 0 | 0.68 | 0.08 | 0 |
| 50 | *LGALS3* | 0.74 | 0 | 0 | 0.98 | 0.62 | 0 |
| 51 | *CCL20* | 1.25 | 1 | 0 | 0.51 | 0.18 | 0 |
| 52 | *CTSB* | 1.17 | 1 | 0 | 0.96 | 0.48 | 0 |
| 53 | *CCL18* | 1.14 | 1 | 0 | 0.60 | 0.31 | 0 |
| 54 | *CTSD* | 1.14 | 1 | 0 | 0.98 | 0.58 | 0 |
| 55 | *APOE* | 1.08 | 1 | 0 | 0.77 | 0.38 | 0 |
| 56 | *CTSL* | 1.06 | 1 | 0 | 0.88 | 0.40 | 0 |
| 57 | *HMOX1* | 1.06 | 1 | 0 | 0.48 | 0.18 | 0 |
| 58 | *RETN* | 1.00 | 1 | 0 | 0.52 | 0.22 | 0 |
| 59 | *OLR1* | 0.99 | 1 | 0 | 0.79 | 0.28 | 0 |
| 60 | *CXCL3* | 0.99 | 1 | 0 | 0.82 | 0.40 | 0 |
| 61 | *MRC1* | 0.96 | 1 | 0 | 0.80 | 0.28 | 0 |
| 62 | *GPNMB* | 0.95 | 1 | 0 | 0.78 | 0.28 | 0 |
| 63 | *CD163* | 0.93 | 1 | 0 | 0.76 | 0.27 | 0 |
| 64 | *MARCO* | 0.87 | 1 | 0 | 0.81 | 0.31 | 0 |
| 65 | *ACP5* | 0.85 | 1 | 0 | 0.88 | 0.36 | 0 |
| 66 | *GPX1* | 0.85 | 1 | 0 | 0.98 | 0.63 | 0 |
| 67 | *LYZ* | 0.83 | 1 | 0 | 0.89 | 0.47 | 0 |
| 68 | *APOC1* | 0.82 | 1 | 0 | 0.88 | 0.47 | 0 |
| 69 | *PSAP* | 0.82 | 1 | 0 | 0.97 | 0.59 | 0 |
| 70 | *CTSZ* | 0.81 | 1 | 0 | 0.76 | 0.32 | 0 |
| 71 | *LGALS1* | 0.79 | 1 | 0 | 0.98 | 0.66 | 0 |
| 72 | *CD68* | 0.78 | 1 | 0 | 0.89 | 0.39 | 0 |
| 73 | *AP2S1* | 0.78 | 1 | 0 | 0.91 | 0.49 | 0 |
| 74 | *GLUL* | 0.78 | 1 | 0 | 0.96 | 0.58 | 0 |
| 75 | *AGRP* | 0.77 | 1 | 0 | 0.38 | 0.12 | 0 |
| 76 | *FTL* | 0.77 | 1 | 0 | 1.00 | 0.99 | 0 |
| 77 | *GRN* | 0.77 | 1 | 0 | 0.89 | 0.47 | 0 |
| 78 | *MS4A7* | 0.76 | 1 | 0 | 0.88 | 0.35 | 0 |
| 79 | *VSIG4* | 0.76 | 1 | 0 | 0.78 | 0.30 | 0 |
| 80 | *MMP19* | 0.74 | 1 | 0 | 0.66 | 0.25 | 0 |
| 81 | *H2AFY* | 0.73 | 1 | 0 | 0.87 | 0.44 | 0 |
| 82 | *MS4A6A* | 0.73 | 1 | 0 | 0.64 | 0.22 | 0 |
| 83 | *GCHFR* | 0.72 | 1 | 0 | 0.80 | 0.39 | 0 |
| 84 | *FCER1G* | 0.72 | 1 | 0 | 0.97 | 0.51 | 0 |
| 85 | *KCNMA1* | 0.70 | 1 | 0 | 0.44 | 0.14 | 0 |
| 86 | *CSTB* | 0.69 | 1 | 0 | 0.97 | 0.65 | 0 |

| | | | | | | | |
|---|---|---|---|---|---|---|---|
| 87 | *MS4A4A* | 0.69 | 1 | 0 | 0.68 | 0.26 | 0 |
| 88 | *BLVRB* | 0.68 | 1 | 0 | 0.74 | 0.34 | 0 |
| 89 | *EMP3* | 0.68 | 1 | 0 | 0.90 | 0.53 | 0 |
| 90 | *ATP6V1F* | 0.67 | 1 | 0 | 0.93 | 0.56 | 0 |
| 91 | *CYBB* | 0.67 | 1 | 0 | 0.63 | 0.23 | 0 |
| 92 | *C5AR1* | 0.65 | 1 | 0 | 0.74 | 0.28 | 0 |
| 93 | *THBD* | 0.65 | 1 | 0 | 0.70 | 0.32 | 0 |
| 94 | *BASP1* | 0.65 | 1 | 0 | 0.52 | 0.14 | 0 |
| 95 | *FCGRT* | 0.64 | 1 | 0 | 0.85 | 0.47 | 0 |
| 96 | *PLAUR* | 0.64 | 1 | 0 | 0.87 | 0.41 | 0 |
| 97 | *SLC11A1* | 0.64 | 1 | 0 | 0.77 | 0.32 | 0 |
| 98 | *ATP6V0B* | 0.62 | 1 | 0 | 0.91 | 0.53 | 0 |
| 99 | *MAP3K8* | 0.62 | 1 | 0 | 0.69 | 0.33 | 0 |
| 100 | *PLIN2* | 0.62 | 1 | 0 | 0.86 | 0.47 | 0 |
| 101 | *CXCR4* | 2.20 | 2 | 0 | 0.91 | 0.33 | 0 |
| 102 | *CCL5* | 2.19 | 2 | 0 | 0.60 | 0.05 | 0 |
| 103 | *ZFP36L2* | 1.98 | 2 | 0 | 0.89 | 0.53 | 0 |
| 104 | *TRAC* | 1.94 | 2 | 0 | 0.54 | 0.02 | 0 |
| 105 | *CD3D* | 1.91 | 2 | 0 | 0.60 | 0.03 | 0 |
| 106 | *CCL4* | 1.81 | 2 | 0 | 0.49 | 0.18 | 0 |
| 107 | *CD2* | 1.75 | 2 | 0 | 0.53 | 0.03 | 0 |
| 108 | *TRBC2* | 1.68 | 2 | 0 | 0.46 | 0.02 | 0 |
| 109 | *RGS1* | 1.67 | 2 | 0 | 0.61 | 0.21 | 0 |
| 110 | *TSC22D3* | 1.65 | 2 | 0 | 0.85 | 0.57 | 0 |
| 111 | *IL7R* | 1.54 | 2 | 0 | 0.54 | 0.26 | 0 |
| 112 | *IL32* | 1.54 | 2 | 0 | 0.62 | 0.13 | 0 |
| 113 | *KLRB1* | 1.52 | 2 | 0 | 0.31 | 0.04 | 0 |
| 114 | *TRBC1* | 1.51 | 2 | 0 | 0.36 | 0.03 | 0 |
| 115 | *CD69* | 1.44 | 2 | 0 | 0.62 | 0.11 | 0 |
| 116 | *LTB* | 1.41 | 2 | 0 | 0.31 | 0.05 | 0 |
| 117 | *CD7* | 1.41 | 2 | 0 | 0.48 | 0.05 | 0 |
| 118 | *BTG1* | 1.38 | 2 | 0 | 0.96 | 0.77 | 0 |
| 119 | *PTPRC* | 1.35 | 2 | 0 | 0.64 | 0.36 | 0 |
| 120 | *GPR171* | 1.35 | 2 | 0 | 0.30 | 0.01 | 0 |
| 121 | *CD3E* | 1.31 | 2 | 0 | 0.39 | 0.01 | 0 |
| 122 | *DUSP2* | 1.31 | 2 | 0 | 0.59 | 0.24 | 0 |
| 123 | *ISG20* | 1.27 | 2 | 0 | 0.58 | 0.18 | 0 |
| 124 | *GZMA* | 1.25 | 2 | 0 | 0.33 | 0.04 | 0 |
| 125 | *CLEC2D* | 1.24 | 2 | 0 | 0.35 | 0.04 | 0 |
| 126 | *SYTL3* | 1.24 | 2 | 0 | 0.43 | 0.08 | 0 |
| 127 | *CST7* | 1.21 | 2 | 0 | 0.45 | 0.07 | 0 |
| 128 | *RORA* | 1.21 | 2 | 0 | 0.36 | 0.08 | 0 |
| 129 | *ACAP1* | 1.21 | 2 | 0 | 0.37 | 0.04 | 0 |
| 130 | *CYTIP* | 1.20 | 2 | 0 | 0.54 | 0.29 | 0 |

| | | | | | | | |
|---|---|---|---|---|---|---|---|
| 131 | *TUBA4A* | 1.20 | 2 | 0 | 0.38 | 0.08 | 0 |
| 132 | *STK4* | 1.19 | 2 | 0 | 0.46 | 0.21 | 0 |
| 133 | *LEPROTL1* | 1.18 | 2 | 0 | 0.56 | 0.36 | 0 |
| 134 | *SARAF* | 1.16 | 2 | 0 | 0.84 | 0.67 | 0 |
| 135 | *PPP2R5C* | 1.15 | 2 | 0 | 0.48 | 0.28 | 0 |
| 136 | *RHOH* | 1.13 | 2 | 0 | 0.39 | 0.12 | 0 |
| 137 | *GZMB* | 1.13 | 2 | 0 | 0.33 | 0.06 | 0 |
| 138 | *LCK* | 1.12 | 2 | 0 | 0.34 | 0.02 | 0 |
| 139 | *FYN* | 1.11 | 2 | 0 | 0.38 | 0.08 | 0 |
| 140 | *DUSP4* | 1.11 | 2 | 0 | 0.32 | 0.11 | 0 |
| 141 | *SOCS1* | 1.09 | 2 | 0 | 0.41 | 0.15 | 0 |
| 142 | *CREM* | 1.08 | 2 | 0 | 0.67 | 0.44 | 0 |
| 143 | *SPOCK2* | 1.08 | 2 | 0 | 0.33 | 0.03 | 0 |
| 144 | *CORO1A* | 1.07 | 2 | 0 | 0.49 | 0.19 | 0 |
| 145 | *RP11-138A9.1* | 1.06 | 2 | 0 | 0.32 | 0.12 | 0 |
| 146 | *NKG7* | 1.05 | 2 | 0 | 0.34 | 0.09 | 0 |
| 147 | *DDIT4* | 1.05 | 2 | 0 | 0.62 | 0.34 | 0 |
| 148 | *PRDM1* | 1.02 | 2 | 0 | 0.29 | 0.08 | 0 |
| 149 | *CNOT6L* | 1.01 | 2 | 0 | 0.33 | 0.11 | 0 |
| 150 | *HMGB2* | 1.08 | 2 | ######## | 0.49 | 0.37 | ######## |
| 151 | *FCN3* | 2.86 | 3 | 0 | 0.41 | 0.03 | 0 |
| 152 | *CLDN5* | 2.81 | 3 | 0 | 0.77 | 0.05 | 0 |
| 153 | *ACKR1* | 2.62 | 3 | 0 | 0.37 | 0.02 | 0 |
| 154 | *SPARCL1* | 2.48 | 3 | 0 | 0.79 | 0.08 | 0 |
| 155 | *TM4SF1* | 2.47 | 3 | 0 | 0.80 | 0.12 | 0 |
| 156 | *VWF* | 2.44 | 3 | 0 | 0.67 | 0.02 | 0 |
| 157 | *IFI27* | 2.38 | 3 | 0 | 0.93 | 0.21 | 0 |
| 158 | *EPAS1* | 2.34 | 3 | 0 | 0.82 | 0.24 | 0 |
| 159 | *MT1M* | 2.31 | 3 | 0 | 0.63 | 0.11 | 0 |
| 160 | *IL6* | 2.20 | 3 | 0 | 0.41 | 0.08 | 0 |
| 161 | *EMP1* | 2.13 | 3 | 0 | 0.74 | 0.23 | 0 |
| 162 | *RAMP2* | 2.09 | 3 | 0 | 0.67 | 0.03 | 0 |
| 163 | *HYAL2* | 2.09 | 3 | 0 | 0.68 | 0.09 | 0 |
| 164 | *GNG11* | 2.04 | 3 | 0 | 0.72 | 0.08 | 0 |
| 165 | *AQP1* | 2.04 | 3 | 0 | 0.64 | 0.05 | 0 |
| 166 | *MT1X* | 2.00 | 3 | 0 | 0.68 | 0.29 | 0 |
| 167 | *MT1A* | 1.99 | 3 | 0 | 0.39 | 0.06 | 0 |
| 168 | *CCL2* | 1.97 | 3 | 0 | 0.56 | 0.18 | 0 |
| 169 | *TIMP3* | 1.97 | 3 | 0 | 0.72 | 0.10 | 0 |
| 170 | *SOCS3* | 1.97 | 3 | 0 | 0.82 | 0.46 | 0 |
| 171 | *CLEC14A* | 1.94 | 3 | 0 | 0.60 | 0.01 | 0 |
| 172 | *IFITM3* | 1.87 | 3 | 0 | 0.92 | 0.46 | 0 |
| 173 | *EGFL7* | 1.83 | 3 | 0 | 0.60 | 0.08 | 0 |
| 174 | *PRSS23* | 1.82 | 3 | 0 | 0.58 | 0.05 | 0 |

| | | | | | | | |
|---|---|---|---|---|---|---|---|
| 175 | *FAM107A* | 1.81 | 3 | 0 | 0.55 | 0.01 | 0 |
| 176 | *MT2A* | 1.80 | 3 | 0 | 0.92 | 0.64 | 0 |
| 177 | *TMEM100* | 1.79 | 3 | 0 | 0.30 | 0.02 | 0 |
| 178 | *CSF3* | 1.78 | 3 | 0 | 0.31 | 0.03 | 0 |
| 179 | *ID1* | 1.77 | 3 | 0 | 0.57 | 0.12 | 0 |
| 180 | *IGFBP7* | 1.75 | 3 | 0 | 0.75 | 0.23 | 0 |
| 181 | *EDN1* | 1.75 | 3 | 0 | 0.34 | 0.03 | 0 |
| 182 | *SLC9A3R2* | 1.73 | 3 | 0 | 0.53 | 0.07 | 0 |
| 183 | *MT1E* | 1.72 | 3 | 0 | 0.59 | 0.24 | 0 |
| 184 | *PDLIM1* | 1.71 | 3 | 0 | 0.75 | 0.32 | 0 |
| 185 | *RAMP3* | 1.70 | 3 | 0 | 0.49 | 0.01 | 0 |
| 186 | *ENG* | 1.70 | 3 | 0 | 0.62 | 0.25 | 0 |
| 187 | *SPARC* | 1.70 | 3 | 0 | 0.63 | 0.13 | 0 |
| 188 | *PECAM1* | 1.70 | 3 | 0 | 0.71 | 0.26 | 0 |
| 189 | *CAV1* | 1.66 | 3 | 0 | 0.72 | 0.10 | 0 |
| 190 | *NPDC1* | 1.65 | 3 | 0 | 0.61 | 0.04 | 0 |
| 191 | *TCF4* | 1.63 | 3 | 0 | 0.60 | 0.07 | 0 |
| 192 | *CLU* | 1.62 | 3 | 0 | 0.44 | 0.12 | 0 |
| 193 | *C10orf10* | 1.62 | 3 | 0 | 0.33 | 0.05 | 0 |
| 194 | *TNFSF10* | 1.61 | 3 | 0 | 0.44 | 0.09 | 0 |
| 195 | *CRIP2* | 1.59 | 3 | 0 | 0.63 | 0.08 | 0 |
| 196 | *ESAM* | 1.57 | 3 | 0 | 0.56 | 0.03 | 0 |
| 197 | *IGFBP4* | 1.51 | 3 | 0 | 0.57 | 0.07 | 0 |
| 198 | *CD59* | 1.51 | 3 | 0 | 0.81 | 0.46 | 0 |
| 199 | *PCAT19* | 1.50 | 3 | 0 | 0.54 | 0.02 | 0 |
| 200 | *ICAM1* | 1.48 | 3 | 0 | 0.55 | 0.19 | 0 |
| 201 | *FTL* | 0.95 | 4 | 0 | 1.00 | 1.00 | 0 |
| 202 | *FTH1* | 0.74 | 4 | 0 | 1.00 | 0.99 | 0 |
| 203 | *YBX1* | 0.71 | 4 | ######## | 0.79 | 0.84 | ######## |
| 204 | *CSTB* | 0.80 | 4 | ######## | 0.73 | 0.71 | ######## |
| 205 | *HLA-DRA* | 0.74 | 4 | ######## | 0.78 | 0.72 | ######## |
| 206 | *LGALS3* | 0.96 | 4 | ######## | 0.67 | 0.69 | ######## |
| 207 | *S100A11* | 0.56 | 4 | ######## | 0.78 | 0.78 | ######## |
| 208 | *VIM* | 0.52 | 4 | ######## | 0.82 | 0.84 | ######## |
| 209 | *APOC1* | 0.60 | 4 | ######## | 0.66 | 0.53 | ######## |
| 210 | *APOE* | 1.34 | 4 | ######## | 0.52 | 0.44 | ######## |
| 211 | *RAB13* | 0.47 | 4 | 2.79E-67 | 0.20 | 0.44 | 9.40E-63 |
| 212 | *FDX1* | 0.51 | 4 | 3.29E-64 | 0.16 | 0.37 | 1.11E-59 |
| 213 | *SNX2* | 0.47 | 4 | 3.29E-61 | 0.16 | 0.36 | 1.11E-56 |
| 214 | *HLA-DPA1* | 0.75 | 4 | 1.31E-59 | 0.59 | 0.63 | 4.42E-55 |
| 215 | *GPX1* | 0.50 | 4 | 1.12E-57 | 0.65 | 0.70 | 3.76E-53 |
| 216 | *CREG1* | 0.50 | 4 | 1.43E-57 | 0.18 | 0.39 | 4.83E-53 |
| 217 | *AKR1B1* | 0.48 | 4 | 6.33E-44 | 0.17 | 0.34 | 2.13E-39 |
| 218 | *HLA-DPB1* | 0.71 | 4 | 2.70E-42 | 0.56 | 0.61 | 9.10E-38 |

| | | | | | | | |
|---|---|---|---|---|---|---|---|
| 219 | *SH3BGRL* | 0.47 | 4 | 8.42E-41 | 0.29 | 0.55 | 2.84E-36 |
| 220 | *CYP27A1* | 0.48 | 4 | 1.12E-40 | 0.17 | 0.34 | 3.76E-36 |
| 221 | *CTSD* | 0.53 | 4 | 9.55E-33 | 0.61 | 0.66 | 3.22E-28 |
| 222 | *MDH1* | 0.65 | 4 | 2.04E-31 | 0.18 | 0.33 | 6.86E-27 |
| 223 | *PYCARD* | 0.49 | 4 | 2.43E-30 | 0.25 | 0.45 | 8.20E-26 |
| 224 | *GCHFR* | 0.84 | 4 | 2.60E-28 | 0.44 | 0.47 | 8.76E-24 |
| 225 | *NCF2* | 0.60 | 4 | 1.10E-27 | 0.20 | 0.36 | 3.69E-23 |
| 226 | *CTSC* | 0.53 | 4 | 1.50E-26 | 0.29 | 0.51 | 5.05E-22 |
| 227 | *FBP1* | 0.85 | 4 | 1.01E-23 | 0.41 | 0.43 | 3.39E-19 |
| 228 | *ARPC3* | 0.61 | 4 | 2.33E-23 | 0.55 | 0.74 | 7.84E-19 |
| 229 | *ARPC5* | 0.52 | 4 | 2.69E-23 | 0.34 | 0.61 | 9.06E-19 |
| 230 | *IGSF6* | 0.63 | 4 | 3.98E-23 | 0.18 | 0.31 | 1.34E-18 |
| 231 | *TALDO1* | 0.66 | 4 | 4.32E-23 | 0.27 | 0.48 | 1.46E-18 |
| 232 | *SLC25A5* | 0.49 | 4 | 6.49E-23 | 0.36 | 0.65 | 2.19E-18 |
| 233 | *NUPR1* | 0.60 | 4 | 1.39E-19 | 0.25 | 0.40 | 4.68E-15 |
| 234 | *ALDH2* | 0.52 | 4 | 3.09E-19 | 0.29 | 0.49 | 1.04E-14 |
| 235 | *CTSL* | 0.61 | 4 | 3.68E-19 | 0.30 | 0.50 | 1.24E-14 |
| 236 | *SPI1* | 0.46 | 4 | 9.80E-18 | 0.25 | 0.41 | 3.30E-13 |
| 237 | *CCL18* | 0.71 | 4 | 5.12E-16 | 0.37 | 0.36 | 1.72E-11 |
| 238 | *HLA-DMA* | 0.48 | 4 | 2.34E-13 | 0.30 | 0.48 | 7.88E-09 |
| 239 | *HLA-DQA1* | 0.50 | 4 | 5.22E-11 | 0.25 | 0.38 | 1.76E-06 |
| 240 | *FABP5* | 0.58 | 4 | 8.12E-10 | 0.46 | 0.55 | 2.73E-05 |
| 241 | *PLIN2* | 0.56 | 4 | 3.39E-07 | 0.36 | 0.55 | 0.011433 |
| 242 | *TSPO* | 0.52 | 4 | 1.46E-06 | 0.50 | 0.67 | 0.049298 |
| 243 | *CD68* | 0.50 | 4 | 1.35E-05 | 0.41 | 0.48 | 0.454645 |
| 244 | *HLA-DQB1* | 0.50 | 4 | 1.65E-05 | 0.33 | 0.48 | 0.55547 |
| 245 | *VAMP8* | 0.48 | 4 | 1.80E-05 | 0.39 | 0.62 | 0.605765 |
| 246 | *ATP6V1F* | 0.55 | 4 | 0.000119 | 0.47 | 0.63 | 1 |
| 247 | *ANXA5* | 0.51 | 4 | 0.000131 | 0.41 | 0.65 | 1 |
| 248 | *VSIG4* | 0.56 | 4 | 0.000459 | 0.29 | 0.40 | 1 |
| 249 | *CAPG* | 0.65 | 4 | 0.004678 | 0.37 | 0.47 | 1 |
| 250 | *PSMA7* | 0.47 | 4 | 0.006263 | 0.45 | 0.71 | 1 |
| 251 | *S100A8* | 3.03 | 5 | 0 | 0.81 | 0.29 | 0 |
| 252 | *S100A9* | 2.65 | 5 | 0 | 0.91 | 0.46 | 0 |
| 253 | *G0S2* | 2.27 | 5 | 0 | 0.69 | 0.16 | 0 |
| 254 | *IL1B* | 2.16 | 5 | 0 | 0.69 | 0.21 | 0 |
| 255 | *S100A12* | 2.11 | 5 | 0 | 0.40 | 0.01 | 0 |
| 256 | *FCN1* | 2.09 | 5 | 0 | 0.74 | 0.07 | 0 |
| 257 | *THBS1* | 2.04 | 5 | 0 | 0.76 | 0.32 | 0 |
| 258 | *SERPINB2* | 2.00 | 5 | 0 | 0.33 | 0.02 | 0 |
| 259 | *EREG* | 1.99 | 5 | 0 | 0.73 | 0.17 | 0 |
| 260 | *VCAN* | 1.82 | 5 | 0 | 0.64 | 0.16 | 0 |
| 261 | *TIMP1* | 1.74 | 5 | 0 | 0.95 | 0.51 | 0 |
| 262 | *NAMPT* | 1.65 | 5 | 0 | 0.92 | 0.53 | 0 |

| | | | | | | | |
|---|---|---|---|---|---|---|---|
| 263 | *PLAUR* | 1.63 | 5 | 0 | 0.91 | 0.46 | 0 |
| 264 | *CD300E* | 1.52 | 5 | 0 | 0.61 | 0.06 | 0 |
| 265 | *LST1* | 1.44 | 5 | 0 | 0.84 | 0.39 | 0 |
| 266 | *RP11-1143G9.4* | 1.36 | 5 | 0 | 0.60 | 0.26 | 0 |
| 267 | *SOD2* | 1.35 | 5 | 0 | 0.90 | 0.54 | 0 |
| 268 | *AIF1* | 1.35 | 5 | 0 | 0.90 | 0.45 | 0 |
| 269 | *C5AR1* | 1.33 | 5 | 0 | 0.78 | 0.33 | 0 |
| 270 | *PPIF* | 1.32 | 5 | 0 | 0.54 | 0.19 | 0 |
| 271 | *IL1R2* | 1.27 | 5 | 0 | 0.35 | 0.04 | 0 |
| 272 | *SAT1* | 1.25 | 5 | 0 | 1.00 | 0.83 | 0 |
| 273 | *APOBEC3A* | 1.24 | 5 | 0 | 0.35 | 0.01 | 0 |
| 274 | *CFP* | 1.24 | 5 | 0 | 0.50 | 0.05 | 0 |
| 275 | *AREG* | 1.18 | 5 | 0 | 0.83 | 0.46 | 0 |
| 276 | *FGL2* | 1.17 | 5 | 0 | 0.49 | 0.07 | 0 |
| 277 | *CD14* | 1.16 | 5 | 0 | 0.63 | 0.24 | 0 |
| 278 | *TYMP* | 1.14 | 5 | 0 | 0.88 | 0.52 | 0 |
| 279 | *BCL2A1* | 1.11 | 5 | 0 | 0.58 | 0.31 | 0 |
| 280 | *SLC2A3* | 1.11 | 5 | 0 | 0.72 | 0.38 | 0 |
| 281 | *SAMSN1* | 1.11 | 5 | 0 | 0.80 | 0.38 | 0 |
| 282 | *RGS2* | 1.10 | 5 | 0 | 0.69 | 0.28 | 0 |
| 283 | *CTB-61M7.2* | 1.09 | 5 | 0 | 0.37 | 0.03 | 0 |
| 284 | *COTL1* | 1.08 | 5 | 0 | 0.85 | 0.52 | 0 |
| 285 | *MXD1* | 1.08 | 5 | 0 | 0.57 | 0.20 | 0 |
| 286 | *SRGN* | 1.07 | 5 | 0 | 0.98 | 0.75 | 0 |
| 287 | *NLRP3* | 1.04 | 5 | 0 | 0.39 | 0.07 | 0 |
| 288 | *FPR1* | 1.03 | 5 | 0 | 0.54 | 0.20 | 0 |
| 289 | *INSIG1* | 1.02 | 5 | 0 | 0.53 | 0.24 | 0 |
| 290 | *SERPINB9* | 1.02 | 5 | 0 | 0.50 | 0.09 | 0 |
| 291 | *FCER1G* | 0.99 | 5 | 0 | 0.96 | 0.56 | 0 |
| 292 | *LYZ* | 0.99 | 5 | 0 | 0.91 | 0.51 | 0 |
| 293 | *FPR2* | 0.99 | 5 | 0 | 0.41 | 0.16 | 0 |
| 294 | *BASP1* | 0.98 | 5 | 0 | 0.50 | 0.18 | 0 |
| 295 | *ATP1B3* | 0.98 | 5 | 0 | 0.80 | 0.55 | 0 |
| 296 | *LILRA5* | 0.94 | 5 | 0 | 0.35 | 0.04 | 0 |
| 297 | *OSM* | 0.92 | 5 | 0 | 0.33 | 0.10 | 0 |
| 298 | *RILPL2* | 0.91 | 5 | 0 | 0.64 | 0.37 | 0 |
| 299 | *CXCL8* | 1.44 | 5 | ######## | 0.59 | 0.31 | ######## |
| 300 | *C15orf48* | 1.22 | 5 | 1.12E-97 | 0.33 | 0.19 | 3.77E-93 |
| 301 | *SFTPC* | 5.58 | 6 | 0 | 0.98 | 0.37 | 0 |
| 302 | *SFTPA1* | 4.55 | 6 | 0 | 0.97 | 0.09 | 0 |
| 303 | *SFTPA2* | 4.41 | 6 | 0 | 0.95 | 0.08 | 0 |
| 304 | *SFTPB* | 3.71 | 6 | 0 | 0.98 | 0.11 | 0 |
| 305 | *NAPSA* | 3.10 | 6 | 0 | 0.91 | 0.04 | 0 |
| 306 | *SFTPD* | 2.70 | 6 | 0 | 0.86 | 0.02 | 0 |

| | | | | | | | |
|---|---|---|---|---|---|---|---|
| 307 | *PGC* | 2.61 | 6 | 0 | 0.74 | 0.02 | 0 |
| 308 | *SLPI* | 2.55 | 6 | 0 | 0.95 | 0.14 | 0 |
| 309 | *SFTA2* | 2.43 | 6 | 0 | 0.88 | 0.04 | 0 |
| 310 | *SLC34A2* | 2.22 | 6 | 0 | 0.79 | 0.03 | 0 |
| 311 | *SFTA3* | 1.89 | 6 | 0 | 0.79 | 0.03 | 0 |
| 312 | *CYB5A* | 1.86 | 6 | 0 | 0.93 | 0.44 | 0 |
| 313 | *MUC1* | 1.85 | 6 | 0 | 0.77 | 0.04 | 0 |
| 314 | *CXCL17* | 1.85 | 6 | 0 | 0.78 | 0.03 | 0 |
| 315 | *S100A14* | 1.84 | 6 | 0 | 0.76 | 0.02 | 0 |
| 316 | *HOPX* | 1.66 | 6 | 0 | 0.84 | 0.14 | 0 |
| 317 | *PEBP4* | 1.62 | 6 | 0 | 0.68 | 0.02 | 0 |
| 318 | *AK1* | 1.61 | 6 | 0 | 0.75 | 0.11 | 0 |
| 319 | *NPC2* | 1.61 | 6 | 0 | 0.97 | 0.67 | 0 |
| 320 | *PIGR* | 1.58 | 6 | 0 | 0.67 | 0.03 | 0 |
| 321 | *C8orf4* | 1.51 | 6 | 0 | 0.66 | 0.09 | 0 |
| 322 | *ABCA3* | 1.47 | 6 | 0 | 0.67 | 0.01 | 0 |
| 323 | *CTSH* | 1.47 | 6 | 0 | 0.80 | 0.37 | 0 |
| 324 | *LAMP3* | 1.41 | 6 | 0 | 0.65 | 0.01 | 0 |
| 325 | *KRT18* | 1.40 | 6 | 0 | 0.72 | 0.09 | 0 |
| 326 | *C16orf89* | 1.40 | 6 | 0 | 0.67 | 0.02 | 0 |
| 327 | *MGST1* | 1.38 | 6 | 0 | 0.82 | 0.32 | 0 |
| 328 | *MALL* | 1.38 | 6 | 0 | 0.67 | 0.03 | 0 |
| 329 | *LRRK2* | 1.34 | 6 | 0 | 0.62 | 0.04 | 0 |
| 330 | *LPCAT1* | 1.29 | 6 | 0 | 0.69 | 0.09 | 0 |
| 331 | *ELF3* | 1.29 | 6 | 0 | 0.67 | 0.04 | 0 |
| 332 | *C11orf96* | 1.28 | 6 | 0 | 0.62 | 0.06 | 0 |
| 333 | *SDC4* | 1.24 | 6 | 0 | 0.67 | 0.24 | 0 |
| 334 | *AQP3* | 1.23 | 6 | 0 | 0.70 | 0.23 | 0 |
| 335 | *WIF1* | 1.20 | 6 | 0 | 0.53 | 0.01 | 0 |
| 336 | *KRT8* | 1.18 | 6 | 0 | 0.66 | 0.07 | 0 |
| 337 | *SDR16C5* | 1.17 | 6 | 0 | 0.56 | 0.01 | 0 |
| 338 | *SELENBP1* | 1.17 | 6 | 0 | 0.61 | 0.03 | 0 |
| 339 | *SLC39A8* | 1.14 | 6 | 0 | 0.66 | 0.08 | 0 |
| 340 | *DMBT1* | 1.14 | 6 | 0 | 0.39 | 0.00 | 0 |
| 341 | *C4BPA* | 1.12 | 6 | 0 | 0.54 | 0.01 | 0 |
| 342 | *AGR2* | 1.12 | 6 | 0 | 0.55 | 0.04 | 0 |
| 343 | *SCGB3A2* | 1.10 | 6 | 0 | 0.53 | 0.08 | 0 |
| 344 | *SLC6A14* | 1.10 | 6 | 0 | 0.46 | 0.01 | 0 |
| 345 | *MID1IP1* | 1.09 | 6 | 0 | 0.59 | 0.15 | 0 |
| 346 | *DHCR24* | 1.08 | 6 | 0 | 0.61 | 0.07 | 0 |
| 347 | *FOLR1* | 1.08 | 6 | 0 | 0.62 | 0.06 | 0 |
| 348 | *CLDN18* | 1.08 | 6 | 0 | 0.55 | 0.01 | 0 |
| 349 | *MRPL14* | 1.07 | 6 | 0 | 0.75 | 0.36 | 0 |
| 350 | *CXCL2* | 1.10 | 6 | ######## | 0.74 | 0.44 | ######## |

| | | | | | | | |
|---|---|---|---|---|---|---|---|
| 351 | *DCN* | 4.10 | 7 | 0 | 0.93 | 0.07 | 0 |
| 352 | *LUM* | 3.54 | 7 | 0 | 0.77 | 0.02 | 0 |
| 353 | *FBLN1* | 3.16 | 7 | 0 | 0.73 | 0.02 | 0 |
| 354 | *CFD* | 2.85 | 7 | 0 | 0.81 | 0.36 | 0 |
| 355 | *A2M* | 2.82 | 7 | 0 | 0.69 | 0.15 | 0 |
| 356 | *MGP* | 2.78 | 7 | 0 | 0.85 | 0.14 | 0 |
| 357 | *RARRES2* | 2.70 | 7 | 0 | 0.74 | 0.05 | 0 |
| 358 | *PTGDS* | 2.65 | 7 | 0 | 0.55 | 0.05 | 0 |
| 359 | *ADH1B* | 2.60 | 7 | 0 | 0.68 | 0.02 | 0 |
| 360 | *MFAP4* | 2.60 | 7 | 0 | 0.67 | 0.02 | 0 |
| 361 | *IGFBP6* | 2.45 | 7 | 0 | 0.37 | 0.03 | 0 |
| 362 | *C1R* | 2.44 | 7 | 0 | 0.67 | 0.05 | 0 |
| 363 | *C1S* | 2.43 | 7 | 0 | 0.66 | 0.03 | 0 |
| 364 | *CYR61* | 2.39 | 7 | 0 | 0.59 | 0.09 | 0 |
| 365 | *COL1A2* | 2.29 | 7 | 0 | 0.57 | 0.01 | 0 |
| 366 | *PTX3* | 2.24 | 7 | 0 | 0.26 | 0.03 | 0 |
| 367 | *GPX3* | 2.22 | 7 | 0 | 0.80 | 0.29 | 0 |
| 368 | *APOD* | 2.15 | 7 | 0 | 0.39 | 0.02 | 0 |
| 369 | *SERPINF1* | 2.15 | 7 | 0 | 0.52 | 0.08 | 0 |
| 370 | *SEPP1* | 2.14 | 7 | 0 | 0.72 | 0.14 | 0 |
| 371 | *GPC3* | 2.03 | 7 | 0 | 0.48 | 0.01 | 0 |
| 372 | *NBL1* | 1.96 | 7 | 0 | 0.60 | 0.04 | 0 |
| 373 | *COL6A2* | 1.96 | 7 | 0 | 0.59 | 0.03 | 0 |
| 374 | *CTGF* | 1.91 | 7 | 0 | 0.48 | 0.07 | 0 |
| 375 | *TCF21* | 1.88 | 7 | 0 | 0.44 | 0.01 | 0 |
| 376 | *PRELP* | 1.87 | 7 | 0 | 0.51 | 0.01 | 0 |
| 377 | *C7* | 1.86 | 7 | 0 | 0.47 | 0.02 | 0 |
| 378 | *CCDC80* | 1.86 | 7 | 0 | 0.35 | 0.02 | 0 |
| 379 | *SERPING1* | 1.84 | 7 | 0 | 0.72 | 0.28 | 0 |
| 380 | *RARRES1* | 1.81 | 7 | 0 | 0.33 | 0.04 | 0 |
| 381 | *NNMT* | 1.79 | 7 | 0 | 0.61 | 0.16 | 0 |
| 382 | *FMO2* | 1.77 | 7 | 0 | 0.45 | 0.02 | 0 |
| 383 | *MMP2* | 1.74 | 7 | 0 | 0.51 | 0.02 | 0 |
| 384 | *TIMP3* | 1.74 | 7 | 0 | 0.67 | 0.13 | 0 |
| 385 | *C11orf96* | 1.72 | 7 | 0 | 0.50 | 0.08 | 0 |
| 386 | *COL3A1* | 1.72 | 7 | 0 | 0.34 | 0.01 | 0 |
| 387 | *PLAC9* | 1.70 | 7 | 0 | 0.45 | 0.03 | 0 |
| 388 | *INMT* | 1.59 | 7 | 0 | 0.34 | 0.02 | 0 |
| 389 | *COL6A1* | 1.55 | 7 | 0 | 0.50 | 0.03 | 0 |
| 390 | *PPP1R14A* | 1.55 | 7 | 0 | 0.39 | 0.05 | 0 |
| 391 | *FBLN5* | 1.54 | 7 | 0 | 0.47 | 0.02 | 0 |
| 392 | *COL1A1* | 1.53 | 7 | 0 | 0.35 | 0.01 | 0 |
| 393 | *IGFBP5* | 1.52 | 7 | 0 | 0.31 | 0.03 | 0 |
| 394 | *IGFBP7* | 1.52 | 7 | 0 | 0.66 | 0.26 | 0 |

| | | | | | | | |
|---|---|---|---|---|---|---|---|
| 395 | *FGF7* | 1.49 | 7 | 0 | 0.41 | 0.03 | 0 |
| 396 | *FHL1* | 1.69 | 7 | ######## | 0.57 | 0.23 | ######## |
| 397 | *FN1* | 1.68 | 7 | ######## | 0.60 | 0.24 | ######## |
| 398 | *EGR1* | 1.48 | 7 | ######## | 0.59 | 0.24 | ######## |
| 399 | *CCL2* | 1.49 | 7 | ######## | 0.54 | 0.20 | ######## |
| 400 | *TIMP1* | 1.54 | 7 | ######## | 0.75 | 0.53 | ######## |
| 401 | *GNLY* | 3.11 | 8 | 0 | 0.91 | 0.04 | 0 |
| 402 | *NKG7* | 2.97 | 8 | 0 | 0.98 | 0.10 | 0 |
| 403 | *GZMB* | 2.87 | 8 | 0 | 0.95 | 0.06 | 0 |
| 404 | *CCL4* | 2.62 | 8 | 0 | 0.93 | 0.20 | 0 |
| 405 | *PRF1* | 2.41 | 8 | 0 | 0.83 | 0.04 | 0 |
| 406 | *CCL3* | 2.35 | 8 | 0 | 0.58 | 0.17 | 0 |
| 407 | *FGFBP2* | 2.33 | 8 | 0 | 0.68 | 0.02 | 0 |
| 408 | *CST7* | 2.26 | 8 | 0 | 0.89 | 0.10 | 0 |
| 409 | *KLRD1* | 2.23 | 8 | 0 | 0.76 | 0.04 | 0 |
| 410 | *CD7* | 2.19 | 8 | 0 | 0.83 | 0.09 | 0 |
| 411 | *KLRB1* | 2.02 | 8 | 0 | 0.65 | 0.06 | 0 |
| 412 | *CCL5* | 1.92 | 8 | 0 | 0.76 | 0.11 | 0 |
| 413 | *CTSW* | 1.91 | 8 | 0 | 0.67 | 0.09 | 0 |
| 414 | *SPON2* | 1.91 | 8 | 0 | 0.63 | 0.04 | 0 |
| 415 | *GZMA* | 1.87 | 8 | 0 | 0.70 | 0.06 | 0 |
| 416 | *KLRF1* | 1.86 | 8 | 0 | 0.54 | 0.01 | 0 |
| 417 | *CD247* | 1.81 | 8 | 0 | 0.63 | 0.04 | 0 |
| 418 | *DUSP2* | 1.74 | 8 | 0 | 0.81 | 0.27 | 0 |
| 419 | *GZMH* | 1.66 | 8 | 0 | 0.50 | 0.03 | 0 |
| 420 | *SYTL3* | 1.61 | 8 | 0 | 0.62 | 0.12 | 0 |
| 421 | *GZMM* | 1.51 | 8 | 0 | 0.51 | 0.04 | 0 |
| 422 | *XCL2* | 1.41 | 8 | 0 | 0.28 | 0.02 | 0 |
| 423 | *CXCR4* | 1.41 | 8 | 0 | 0.90 | 0.39 | 0 |
| 424 | *ISG20* | 1.37 | 8 | 0 | 0.69 | 0.22 | 0 |
| 425 | *DDIT4* | 1.25 | 8 | 0 | 0.76 | 0.37 | 0 |
| 426 | *SH2D2A* | 1.23 | 8 | 0 | 0.37 | 0.03 | 0 |
| 427 | *AKNA* | 1.23 | 8 | 0 | 0.42 | 0.09 | 0 |
| 428 | *CD69* | 1.22 | 8 | 0 | 0.68 | 0.16 | 0 |
| 429 | *TRBC1* | 1.22 | 8 | 0 | 0.44 | 0.06 | 0 |
| 430 | *S1PR5* | 1.22 | 8 | 0 | 0.32 | 0.01 | 0 |
| 431 | *CLIC3* | 1.18 | 8 | 0 | 0.39 | 0.06 | 0 |
| 432 | *SLA2* | 1.17 | 8 | 0 | 0.34 | 0.02 | 0 |
| 433 | *PYHIN1* | 1.07 | 8 | 0 | 0.28 | 0.02 | 0 |
| 434 | *PTPN7* | 1.07 | 8 | 0 | 0.35 | 0.06 | 0 |
| 435 | *MATK* | 1.03 | 8 | 0 | 0.32 | 0.06 | 0 |
| 436 | *IL2RB* | 1.03 | 8 | 0 | 0.28 | 0.02 | 0 |
| 437 | *GNG2* | 1.02 | 8 | 0 | 0.33 | 0.05 | 0 |
| 438 | *BTG1* | 0.99 | 8 | 0 | 0.97 | 0.79 | 0 |

| | | | | | | | |
|---|---|---|---|---|---|---|---|
| 439 | *LIMD2* | 1.12 | 8 | ######## | 0.41 | 0.10 | ######## |
| 440 | *CREM* | 1.28 | 8 | ######## | 0.79 | 0.46 | ######## |
| 441 | *CCL4L2* | 1.69 | 8 | ######## | 0.36 | 0.08 | ######## |
| 442 | *PLAC8* | 1.17 | 8 | ######## | 0.53 | 0.19 | ######## |
| 443 | *PIK3R1* | 1.14 | 8 | ######## | 0.40 | 0.12 | ######## |
| 444 | *CORO1A* | 1.00 | 8 | ######## | 0.54 | 0.22 | ######## |
| 445 | *ID2* | 1.12 | 8 | ######## | 0.73 | 0.50 | ######## |
| 446 | *BIN2* | 1.15 | 8 | ######## | 0.42 | 0.17 | ######## |
| 447 | *FCGR3A* | 1.10 | 8 | ######## | 0.63 | 0.38 | ######## |
| 448 | *CHST12* | 1.00 | 8 | ######## | 0.36 | 0.14 | ######## |
| 449 | *PPP2R5C* | 1.01 | 8 | ######## | 0.51 | 0.30 | ######## |
| 450 | *CMC1* | 1.56 | 8 | ######## | 0.42 | 0.24 | ######## |
| 451 | *AGER* | 3.91 | 9 | 0 | 0.82 | 0.04 | 0 |
| 452 | *EMP2* | 3.50 | 9 | 0 | 0.81 | 0.15 | 0 |
| 453 | *KRT7* | 3.06 | 9 | 0 | 0.77 | 0.06 | 0 |
| 454 | *HOPX* | 3.05 | 9 | 0 | 0.88 | 0.16 | 0 |
| 455 | *CEACAM6* | 2.66 | 9 | 0 | 0.57 | 0.02 | 0 |
| 456 | *KRT19* | 2.57 | 9 | 0 | 0.68 | 0.08 | 0 |
| 457 | *CAV1* | 2.52 | 9 | 0 | 0.75 | 0.14 | 0 |
| 458 | *ADIRF* | 2.26 | 9 | 0 | 0.69 | 0.16 | 0 |
| 459 | *TSPAN13* | 2.25 | 9 | 0 | 0.60 | 0.08 | 0 |
| 460 | *FXYD3* | 2.24 | 9 | 0 | 0.59 | 0.07 | 0 |
| 461 | *GPRC5A* | 2.21 | 9 | 0 | 0.55 | 0.07 | 0 |
| 462 | *SFTA2* | 2.13 | 9 | 0 | 0.67 | 0.08 | 0 |
| 463 | *CLIC3* | 2.10 | 9 | 0 | 0.54 | 0.06 | 0 |
| 464 | *CYP4B1* | 2.07 | 9 | 0 | 0.50 | 0.05 | 0 |
| 465 | *LMO7* | 2.04 | 9 | 0 | 0.52 | 0.06 | 0 |
| 466 | *ANXA3* | 2.02 | 9 | 0 | 0.51 | 0.05 | 0 |
| 467 | *TACSTD2* | 2.02 | 9 | 0 | 0.53 | 0.07 | 0 |
| 468 | *TNNC1* | 2.01 | 9 | 0 | 0.46 | 0.01 | 0 |
| 469 | *MYL9* | 1.94 | 9 | 0 | 0.61 | 0.10 | 0 |
| 470 | *KRT18* | 1.92 | 9 | 0 | 0.55 | 0.12 | 0 |
| 471 | *FOLR1* | 1.92 | 9 | 0 | 0.49 | 0.08 | 0 |
| 472 | *CAV2* | 1.89 | 9 | 0 | 0.53 | 0.09 | 0 |
| 473 | *C19orf33* | 1.81 | 9 | 0 | 0.45 | 0.05 | 0 |
| 474 | *CLDN18* | 1.78 | 9 | 0 | 0.43 | 0.04 | 0 |
| 475 | *EPCAM* | 1.74 | 9 | 0 | 0.43 | 0.06 | 0 |
| 476 | *GGTLC1* | 1.73 | 9 | 0 | 0.35 | 0.01 | 0 |
| 477 | *KRT8* | 1.73 | 9 | 0 | 0.50 | 0.10 | 0 |
| 478 | *SFTA1P* | 1.67 | 9 | 0 | 0.38 | 0.04 | 0 |
| 479 | *AQP4* | 1.65 | 9 | 0 | 0.38 | 0.03 | 0 |
| 480 | *NTM* | 1.59 | 9 | 0 | 0.28 | 0.01 | 0 |
| 481 | *CST6* | 1.58 | 9 | 0 | 0.34 | 0.03 | 0 |
| 482 | *SCEL* | 1.50 | 9 | 0 | 0.36 | 0.01 | 0 |

| | | | | | | | |
|---|---|---|---|---|---|---|---|
| 483 | *RTKN2* | 1.42 | 9 | 0 | 0.29 | 0.01 | 0 |
| 484 | *MSLN* | 1.38 | 9 | 0 | 0.28 | 0.01 | 0 |
| 485 | *ANKRD29* | 1.32 | 9 | 0 | 0.33 | 0.03 | 0 |
| 486 | *NAPSA* | 1.31 | 9 | 0 | 0.46 | 0.08 | 0 |
| 487 | *RNASE1* | 1.85 | 9 | ######## | 0.68 | 0.24 | ######## |
| 488 | *SFTPB* | 1.41 | 9 | ######## | 0.57 | 0.14 | ######## |
| 489 | *WFDC2* | 1.32 | 9 | ######## | 0.43 | 0.09 | ######## |
| 490 | *SLC39A8* | 1.62 | 9 | ######## | 0.45 | 0.10 | ######## |
| 491 | *CLDN4* | 1.32 | 9 | ######## | 0.35 | 0.06 | ######## |
| 492 | *NEDD4L* | 1.37 | 9 | ######## | 0.35 | 0.07 | ######## |
| 493 | *CKB* | 1.33 | 9 | ######## | 0.37 | 0.08 | ######## |
| 494 | *DSTN* | 1.70 | 9 | ######## | 0.75 | 0.52 | ######## |
| 495 | *CD55* | 1.71 | 9 | ######## | 0.63 | 0.45 | ######## |
| 496 | *NGFRAP1* | 1.40 | 9 | ######## | 0.46 | 0.20 | ######## |
| 497 | *TNFRSF12A* | 1.34 | 9 | 6.22E-92 | 0.40 | 0.17 | 2.10E-87 |
| 498 | *CD151* | 1.44 | 9 | 5.05E-78 | 0.52 | 0.34 | 1.70E-73 |
| 499 | *RARRES3* | 1.33 | 9 | 1.46E-65 | 0.40 | 0.20 | 4.93E-61 |
| 500 | *RAB11FIP1* | 1.35 | 9 | 5.78E-57 | 0.45 | 0.30 | 1.95E-52 |
| 501 | *TPSAB1* | 5.17 | 10 | 0 | 0.99 | 0.02 | 0 |
| 502 | *TPSB2* | 4.66 | 10 | 0 | 0.98 | 0.01 | 0 |
| 503 | *CD69* | 2.82 | 10 | 0 | 0.93 | 0.17 | 0 |
| 504 | *CPA3* | 2.78 | 10 | 0 | 0.78 | 0.00 | 0 |
| 505 | *HPGDS* | 2.48 | 10 | 0 | 0.75 | 0.05 | 0 |
| 506 | *MS4A2* | 2.19 | 10 | 0 | 0.68 | 0.00 | 0 |
| 507 | *RGS13* | 2.08 | 10 | 0 | 0.61 | 0.01 | 0 |
| 508 | *GATA2* | 1.95 | 10 | 0 | 0.56 | 0.03 | 0 |
| 509 | *HPGD* | 1.89 | 10 | 0 | 0.68 | 0.22 | 0 |
| 510 | *RGS1* | 1.89 | 10 | 0 | 0.79 | 0.26 | 0 |
| 511 | *SLC18A2* | 1.75 | 10 | 0 | 0.51 | 0.00 | 0 |
| 512 | *C1orf186* | 1.68 | 10 | 0 | 0.49 | 0.00 | 0 |
| 513 | *KIT* | 1.67 | 10 | 0 | 0.52 | 0.00 | 0 |
| 514 | *VWA5A* | 1.56 | 10 | 0 | 0.48 | 0.07 | 0 |
| 515 | *MAOB* | 1.46 | 10 | 0 | 0.45 | 0.01 | 0 |
| 516 | *HDC* | 1.45 | 10 | 0 | 0.38 | 0.00 | 0 |
| 517 | *LTC4S* | 1.44 | 10 | 0 | 0.48 | 0.04 | 0 |
| 518 | *IL1RL1* | 1.38 | 10 | 0 | 0.46 | 0.02 | 0 |
| 519 | *RP11-354E11.2* | 1.32 | 10 | 0 | 0.39 | 0.00 | 0 |
| 520 | *FCER1A* | 1.13 | 10 | 0 | 0.28 | 0.01 | 0 |
| 521 | *PTGS1* | 0.99 | 10 | 0 | 0.29 | 0.03 | 0 |
| 522 | *CALB2* | 0.98 | 10 | 0 | 0.27 | 0.00 | 0 |
| 523 | *GPR65* | 1.67 | 10 | ######## | 0.62 | 0.17 | ######## |
| 524 | *AREG* | 1.82 | 10 | ######## | 0.90 | 0.47 | ######## |
| 525 | *BIRC3* | 2.08 | 10 | ######## | 0.70 | 0.25 | ######## |
| 526 | *H3F3B* | 1.11 | 10 | ######## | 0.99 | 0.89 | ######## |

| | | | | | | | |
|---|---|---|---|---|---|---|---|
| 527 | *LAPTM4A* | 1.49 | 10 | ######## | 0.86 | 0.52 | ######## |
| 528 | *RGS2* | 1.77 | 10 | ######## | 0.74 | 0.30 | ######## |
| 529 | *SRGN* | 1.13 | 10 | ######## | 0.96 | 0.76 | ######## |
| 530 | *SMYD3* | 0.98 | 10 | ######## | 0.27 | 0.04 | ######## |
| 531 | *LMNA* | 1.44 | 10 | ######## | 0.85 | 0.58 | ######## |
| 532 | *DDIT4* | 1.55 | 10 | ######## | 0.75 | 0.38 | ######## |
| 533 | *BATF* | 1.18 | 10 | ######## | 0.35 | 0.08 | ######## |
| 534 | *SELK* | 1.34 | 10 | ######## | 0.84 | 0.62 | ######## |
| 535 | *NFKBIA* | 1.16 | 10 | ######## | 0.90 | 0.74 | ######## |
| 536 | *CPM* | 1.50 | 10 | ######## | 0.58 | 0.25 | ######## |
| 537 | *FOSB* | 1.17 | 10 | ######## | 0.76 | 0.45 | ######## |
| 538 | *SOCS1* | 1.29 | 10 | ######## | 0.50 | 0.18 | ######## |
| 539 | *CREM* | 1.01 | 10 | ######## | 0.77 | 0.47 | ######## |
| 540 | *GALC* | 1.14 | 10 | ######## | 0.35 | 0.10 | ######## |
| 541 | *CKLF* | 1.13 | 10 | ######## | 0.67 | 0.41 | ######## |
| 542 | *CTD-3252C9.4* | 1.11 | 10 | ######## | 0.53 | 0.22 | ######## |
| 543 | *PPP1R15A* | 1.10 | 10 | ######## | 0.73 | 0.52 | ######## |
| 544 | *NFKBIZ* | 1.14 | 10 | ######## | 0.52 | 0.24 | ######## |
| 545 | *PTGS2* | 1.07 | 10 | ######## | 0.30 | 0.09 | ######## |
| 546 | *ANXA1* | 1.00 | 10 | 1.18E-93 | 0.85 | 0.72 | 3.97E-89 |
| 547 | *DUSP6* | 1.02 | 10 | 8.94E-69 | 0.36 | 0.16 | 3.01E-64 |
| 548 | *SYAP1* | 1.02 | 10 | 9.07E-57 | 0.51 | 0.35 | 3.06E-52 |
| 549 | *ZNF331* | 0.99 | 10 | 4.01E-45 | 0.48 | 0.33 | 1.35E-40 |
| 550 | *TUBA1A* | 1.24 | 10 | 1.09E-20 | 0.43 | 0.35 | 3.66E-16 |
| 551 | *CAPS* | 3.86 | 11 | 0 | 0.98 | 0.03 | 0 |
| 552 | *TPPP3* | 3.78 | 11 | 0 | 0.98 | 0.06 | 0 |
| 553 | *C9orf24* | 3.26 | 11 | 0 | 0.93 | 0.01 | 0 |
| 554 | *C20orf85* | 3.22 | 11 | 0 | 0.91 | 0.01 | 0 |
| 555 | *TMEM190* | 3.17 | 11 | 0 | 0.83 | 0.01 | 0 |
| 556 | *RSPH1* | 2.95 | 11 | 0 | 0.92 | 0.01 | 0 |
| 557 | *TSPAN1* | 2.81 | 11 | 0 | 0.87 | 0.02 | 0 |
| 558 | *FAM183A* | 2.80 | 11 | 0 | 0.88 | 0.01 | 0 |
| 559 | *C1orf194* | 2.77 | 11 | 0 | 0.88 | 0.01 | 0 |
| 560 | *AGR3* | 2.71 | 11 | 0 | 0.83 | 0.05 | 0 |
| 561 | *CETN2* | 2.61 | 11 | 0 | 0.91 | 0.15 | 0 |
| 562 | *C11orf88* | 2.46 | 11 | 0 | 0.84 | 0.00 | 0 |
| 563 | *PIFO* | 2.45 | 11 | 0 | 0.86 | 0.01 | 0 |
| 564 | *C5orf49* | 2.45 | 11 | 0 | 0.85 | 0.01 | 0 |
| 565 | *C9orf116* | 2.36 | 11 | 0 | 0.84 | 0.01 | 0 |
| 566 | *MORN2* | 2.33 | 11 | 0 | 0.86 | 0.09 | 0 |
| 567 | *ELF3* | 2.32 | 11 | 0 | 0.81 | 0.07 | 0 |
| 568 | *GSTA1* | 2.29 | 11 | 0 | 0.68 | 0.01 | 0 |
| 569 | *CAPSL* | 2.23 | 11 | 0 | 0.82 | 0.00 | 0 |
| 570 | *DYNLRB2* | 2.23 | 11 | 0 | 0.80 | 0.01 | 0 |

| | | | | | | | |
|---|---|---|---|---|---|---|---|
| 571 | *C2orf40* | 2.22 | 11 | 0 | 0.70 | 0.01 | 0 |
| 572 | *PRDX5* | 2.20 | 11 | 0 | 0.95 | 0.54 | 0 |
| 573 | *ZMYND10* | 2.19 | 11 | 0 | 0.82 | 0.01 | 0 |
| 574 | *SNTN* | 2.15 | 11 | 0 | 0.78 | 0.00 | 0 |
| 575 | *AGR2* | 2.15 | 11 | 0 | 0.78 | 0.05 | 0 |
| 576 | *MS4A8* | 2.15 | 11 | 0 | 0.79 | 0.01 | 0 |
| 577 | *LRRIQ1* | 2.12 | 11 | 0 | 0.80 | 0.00 | 0 |
| 578 | *ODF3B* | 2.07 | 11 | 0 | 0.84 | 0.19 | 0 |
| 579 | *CYP4B1* | 2.04 | 11 | 0 | 0.72 | 0.05 | 0 |
| 580 | *CLDN4* | 2.00 | 11 | 0 | 0.76 | 0.06 | 0 |
| 581 | *DNAAF1* | 2.00 | 11 | 0 | 0.74 | 0.02 | 0 |
| 582 | *KRT8* | 1.97 | 11 | 0 | 0.82 | 0.09 | 0 |
| 583 | *FXYD3* | 1.96 | 11 | 0 | 0.79 | 0.07 | 0 |
| 584 | *AC013264.2* | 1.96 | 11 | 0 | 0.75 | 0.01 | 0 |
| 585 | *CFAP126* | 1.94 | 11 | 0 | 0.76 | 0.00 | 0 |
| 586 | *FOXJ1* | 1.94 | 11 | 0 | 0.76 | 0.00 | 0 |
| 587 | *CLDN3* | 1.94 | 11 | 0 | 0.76 | 0.03 | 0 |
| 588 | *FAM229B* | 1.94 | 11 | 0 | 0.78 | 0.03 | 0 |
| 589 | *MORN5* | 1.93 | 11 | 0 | 0.76 | 0.00 | 0 |
| 590 | *FABP6* | 1.93 | 11 | 0 | 0.70 | 0.00 | 0 |
| 591 | *GDF15* | 1.92 | 11 | 0 | 0.59 | 0.06 | 0 |
| 592 | *C12orf75* | 1.91 | 11 | 0 | 0.77 | 0.06 | 0 |
| 593 | *WFDC2* | 1.90 | 11 | 0 | 0.78 | 0.08 | 0 |
| 594 | *CCDC78* | 1.90 | 11 | 0 | 0.75 | 0.00 | 0 |
| 595 | *CCDC146* | 1.90 | 11 | 0 | 0.74 | 0.01 | 0 |
| 596 | *MRPS31* | 1.89 | 11 | 0 | 0.75 | 0.07 | 0 |
| 597 | *ROPN1L* | 1.88 | 11 | 0 | 0.76 | 0.00 | 0 |
| 598 | *SMIM22* | 1.88 | 11 | 0 | 0.74 | 0.03 | 0 |
| 599 | *FAM92B* | 1.88 | 11 | 0 | 0.75 | 0.00 | 0 |
| 600 | *OMG* | 1.87 | 11 | 0 | 0.60 | 0.01 | 0 |
| 601 | *CCL17* | 2.98 | 12 | 0 | 0.34 | 0.01 | 0 |
| 602 | *GPR183* | 1.83 | 12 | 0 | 0.92 | 0.20 | 0 |
| 603 | *HLA-DPB1* | 1.75 | 12 | 0 | 0.99 | 0.60 | 0 |
| 604 | *HLA-DQA1* | 1.61 | 12 | 0 | 0.93 | 0.36 | 0 |
| 605 | *F13A1* | 1.21 | 12 | 0 | 0.44 | 0.02 | 0 |
| 606 | *IL1R2* | 1.15 | 12 | 0 | 0.45 | 0.06 | 0 |
| 607 | *FCGR2B* | 1.10 | 12 | 0 | 0.55 | 0.05 | 0 |
| 608 | *S100B* | 1.03 | 12 | 0 | 0.27 | 0.01 | 0 |
| 609 | *CLEC10A* | 1.00 | 12 | 0 | 0.42 | 0.02 | 0 |
| 610 | *RNASE6* | 0.97 | 12 | 0 | 0.66 | 0.15 | 0 |
| 611 | *FGL2* | 0.97 | 12 | 0 | 0.56 | 0.10 | 0 |
| 612 | *CSF2RA* | 0.92 | 12 | 0 | 0.63 | 0.14 | 0 |
| 613 | *PKIB* | 0.91 | 12 | 0 | 0.36 | 0.02 | 0 |
| 614 | *CD74* | 1.51 | 12 | ######## | 1.00 | 0.76 | ######## |

| | | | | | | | |
|---|---|---|---|---|---|---|---|
| 615 | *HLA-DQB1* | 1.55 | 12 | ######## | 0.97 | 0.46 | ######## |
| 616 | *RGS1* | 1.07 | 12 | ######## | 0.86 | 0.26 | ######## |
| 617 | *HLA-DPA1* | 1.55 | 12 | ######## | 0.99 | 0.62 | ######## |
| 618 | *HLA-DRA* | 1.41 | 12 | ######## | 1.00 | 0.72 | ######## |
| 619 | *INSIG1* | 1.66 | 12 | ######## | 0.76 | 0.25 | ######## |
| 620 | *MS4A6A* | 1.24 | 12 | ######## | 0.82 | 0.28 | ######## |
| 621 | *RGS2* | 1.28 | 12 | ######## | 0.84 | 0.30 | ######## |
| 622 | *HLA-DQA2* | 1.42 | 12 | ######## | 0.69 | 0.20 | ######## |
| 623 | *HLA-DRB1* | 1.39 | 12 | ######## | 0.98 | 0.60 | ######## |
| 624 | *DUSP4* | 1.01 | 12 | ######## | 0.59 | 0.14 | ######## |
| 625 | *SERPINB9* | 0.86 | 12 | ######## | 0.54 | 0.11 | ######## |
| 626 | *HLA-DMB* | 1.04 | 12 | ######## | 0.85 | 0.34 | ######## |
| 627 | *FOLR2* | 0.99 | 12 | ######## | 0.26 | 0.03 | ######## |
| 628 | *CD86* | 0.80 | 12 | ######## | 0.55 | 0.15 | ######## |
| 629 | *HLA-DMA* | 0.99 | 12 | ######## | 0.93 | 0.46 | ######## |
| 630 | *CD83* | 1.30 | 12 | ######## | 0.75 | 0.30 | ######## |
| 631 | *MXD1* | 0.78 | 12 | ######## | 0.66 | 0.22 | ######## |
| 632 | *SAT1* | 0.78 | 12 | ######## | 1.00 | 0.84 | ######## |
| 633 | *RGS10* | 0.77 | 12 | ######## | 0.79 | 0.35 | ######## |
| 634 | *C15orf48* | 1.13 | 12 | ######## | 0.59 | 0.20 | ######## |
| 635 | *HLA-DRB5* | 1.27 | 12 | ######## | 0.86 | 0.44 | ######## |
| 636 | *SRGN* | 0.79 | 12 | ######## | 0.99 | 0.76 | ######## |
| 637 | *TYMP* | 0.79 | 12 | ######## | 0.91 | 0.54 | ######## |
| 638 | *FCGR2A* | 0.84 | 12 | ######## | 0.74 | 0.32 | ######## |
| 639 | *REL* | 1.05 | 12 | ######## | 0.77 | 0.39 | ######## |
| 640 | *AREG* | 1.32 | 12 | ######## | 0.82 | 0.48 | ######## |
| 641 | *AXL* | 0.79 | 12 | ######## | 0.49 | 0.16 | ######## |
| 642 | *ATP1B3* | 0.86 | 12 | ######## | 0.89 | 0.56 | ######## |
| 643 | *HERPUD1* | 0.94 | 12 | ######## | 0.81 | 0.47 | ######## |
| 644 | *CST3* | 0.94 | 12 | ######## | 0.97 | 0.70 | ######## |
| 645 | *SGK1* | 0.84 | 12 | ######## | 0.67 | 0.30 | ######## |
| 646 | *EREG* | 0.91 | 12 | 3.24E-89 | 0.50 | 0.20 | 1.09E-84 |
| 647 | *CXCL8* | 1.04 | 12 | 1.51E-61 | 0.59 | 0.33 | 5.08E-57 |
| 648 | *THBS1* | 0.79 | 12 | 9.11E-57 | 0.58 | 0.34 | 3.07E-52 |
| 649 | *G0S2* | 0.81 | 12 | 4.84E-40 | 0.39 | 0.19 | 1.63E-35 |
| 650 | *SEPP1* | 0.98 | 12 | 2.30E-22 | 0.28 | 0.16 | 7.76E-18 |
| 651 | *TAGLN* | 3.95 | 13 | 0 | 0.87 | 0.14 | 0 |
| 652 | *ACTA2* | 3.79 | 13 | 0 | 0.78 | 0.06 | 0 |
| 653 | *TPM2* | 3.45 | 13 | 0 | 0.83 | 0.05 | 0 |
| 654 | *MYL9* | 3.18 | 13 | 0 | 0.81 | 0.10 | 0 |
| 655 | *CALD1* | 2.57 | 13 | 0 | 0.78 | 0.10 | 0 |
| 656 | *BGN* | 2.43 | 13 | 0 | 0.62 | 0.03 | 0 |
| 657 | *MYH11* | 2.37 | 13 | 0 | 0.54 | 0.01 | 0 |
| 658 | *PPP1R14A* | 2.29 | 13 | 0 | 0.64 | 0.05 | 0 |

| | | | | | | | |
|---|---|---|---|---|---|---|---|
| 659 | *DES* | 2.15 | 13 | 0 | 0.37 | 0.00 | 0 |
| 660 | *ACTG2* | 2.12 | 13 | 0 | 0.38 | 0.00 | 0 |
| 661 | *CRYAB* | 2.09 | 13 | 0 | 0.53 | 0.03 | 0 |
| 662 | *NDUFA4L2* | 2.07 | 13 | 0 | 0.41 | 0.01 | 0 |
| 663 | *SOD3* | 2.03 | 13 | 0 | 0.54 | 0.05 | 0 |
| 664 | *COL1A2* | 1.78 | 13 | 0 | 0.37 | 0.03 | 0 |
| 665 | *COX4I2* | 1.78 | 13 | 0 | 0.34 | 0.01 | 0 |
| 666 | *IGFBP5* | 1.76 | 13 | 0 | 0.40 | 0.04 | 0 |
| 667 | *HIGD1B* | 1.68 | 13 | 0 | 0.26 | 0.01 | 0 |
| 668 | *EGFL6* | 1.67 | 13 | 0 | 0.34 | 0.00 | 0 |
| 669 | *SPARCL1* | 1.65 | 13 | 0 | 0.67 | 0.13 | 0 |
| 670 | *CRISPLD2* | 1.59 | 13 | 0 | 0.44 | 0.03 | 0 |
| 671 | *MYLK* | 1.58 | 13 | 0 | 0.49 | 0.03 | 0 |
| 672 | *WISP2* | 1.55 | 13 | 0 | 0.28 | 0.01 | 0 |
| 673 | *COL6A2* | 1.53 | 13 | 0 | 0.47 | 0.04 | 0 |
| 674 | *PLAC9* | 1.53 | 13 | 0 | 0.47 | 0.04 | 0 |
| 675 | *MFAP4* | 1.41 | 13 | 0 | 0.38 | 0.04 | 0 |
| 676 | *MAP1B* | 1.35 | 13 | 0 | 0.37 | 0.02 | 0 |
| 677 | *PDGFRB* | 1.34 | 13 | 0 | 0.36 | 0.01 | 0 |
| 678 | *CNN1* | 1.33 | 13 | 0 | 0.32 | 0.00 | 0 |
| 679 | *COL18A1* | 1.31 | 13 | 0 | 0.37 | 0.04 | 0 |
| 680 | *PLN* | 1.29 | 13 | 0 | 0.27 | 0.00 | 0 |
| 681 | *ID4* | 1.30 | 13 | ######## | 0.34 | 0.04 | ######## |
| 682 | *TPM1* | 2.15 | 13 | ######## | 0.67 | 0.17 | ######## |
| 683 | *IGFBP7* | 2.19 | 13 | ######## | 0.81 | 0.26 | ######## |
| 684 | *MGP* | 1.82 | 13 | ######## | 0.68 | 0.16 | ######## |
| 685 | *ADIRF* | 2.16 | 13 | ######## | 0.67 | 0.17 | ######## |
| 686 | *MFGE8* | 1.63 | 13 | ######## | 0.50 | 0.11 | ######## |
| 687 | *C11orf96* | 1.78 | 13 | ######## | 0.47 | 0.09 | ######## |
| 688 | *PRKCDBP* | 1.47 | 13 | ######## | 0.48 | 0.10 | ######## |
| 689 | *SPARC* | 1.60 | 13 | ######## | 0.61 | 0.16 | ######## |
| 690 | *GPX3* | 1.70 | 13 | ######## | 0.76 | 0.30 | ######## |
| 691 | *LMCD1* | 1.29 | 13 | ######## | 0.35 | 0.06 | ######## |
| 692 | *SELM* | 1.80 | 13 | ######## | 0.66 | 0.25 | ######## |
| 693 | *DSTN* | 1.91 | 13 | ######## | 0.79 | 0.52 | ######## |
| 694 | *CSRP1* | 1.39 | 13 | ######## | 0.49 | 0.19 | ######## |
| 695 | *MT1M* | 1.39 | 13 | ######## | 0.46 | 0.15 | ######## |
| 696 | *HSPB1* | 1.35 | 13 | ######## | 0.76 | 0.55 | 1.50E-96 |
| 697 | *MT1A* | 1.47 | 13 | 5.27E-93 | 0.32 | 0.08 | 1.77E-88 |
| 698 | *CTGF* | 1.39 | 13 | 3.44E-74 | 0.29 | 0.08 | 1.16E-69 |
| 699 | *IGFBP2* | 1.38 | 13 | 6.74E-53 | 0.29 | 0.11 | 2.27E-48 |
| 700 | *PDK4* | 1.33 | 13 | 3.61E-52 | 0.46 | 0.25 | 1.22E-47 |
| 701 | *STMN1* | 1.89 | 14 | 0 | 0.88 | 0.30 | 0 |
| 702 | *KIAA0101* | 1.85 | 14 | 0 | 0.77 | 0.01 | 0 |

| | | | | | | | |
|---|---|---|---|---|---|---|---|
| 703 | *TYMS* | 1.33 | 14 | 0 | 0.61 | 0.01 | 0 |
| 704 | *TK1* | 1.30 | 14 | 0 | 0.65 | 0.02 | 0 |
| 705 | *UBE2C* | 1.27 | 14 | 0 | 0.52 | 0.00 | 0 |
| 706 | *PTTG1* | 1.24 | 14 | 0 | 0.59 | 0.08 | 0 |
| 707 | *RRM2* | 1.08 | 14 | 0 | 0.46 | 0.00 | 0 |
| 708 | *CDKN3* | 0.99 | 14 | 0 | 0.45 | 0.01 | 0 |
| 709 | *MKI67* | 0.94 | 14 | 0 | 0.46 | 0.00 | 0 |
| 710 | *CDK1* | 0.92 | 14 | 0 | 0.45 | 0.01 | 0 |
| 711 | *BIRC5* | 0.89 | 14 | 0 | 0.46 | 0.00 | 0 |
| 712 | *CENPM* | 0.88 | 14 | 0 | 0.52 | 0.02 | 0 |
| 713 | *NUSAP1* | 0.87 | 14 | 0 | 0.48 | 0.02 | 0 |
| 714 | *TMEM106C* | 0.83 | 14 | 0 | 0.56 | 0.08 | 0 |
| 715 | *CDT1* | 0.82 | 14 | 0 | 0.44 | 0.00 | 0 |
| 716 | *CENPW* | 0.79 | 14 | 0 | 0.58 | 0.09 | 0 |
| 717 | *ZWINT* | 0.78 | 14 | 0 | 0.45 | 0.02 | 0 |
| 718 | *GGH* | 0.73 | 14 | 0 | 0.48 | 0.05 | 0 |
| 719 | *CENPF* | 0.71 | 14 | 0 | 0.39 | 0.01 | 0 |
| 720 | *TOP2A* | 0.71 | 14 | 0 | 0.39 | 0.00 | 0 |
| 721 | *SMC2* | 0.66 | 14 | 0 | 0.49 | 0.06 | 0 |
| 722 | *DTYMK* | 0.91 | 14 | ######## | 0.57 | 0.10 | ######## |
| 723 | *H2AFZ* | 1.61 | 14 | ######## | 0.96 | 0.65 | ######## |
| 724 | *CKS1B* | 1.20 | 14 | ######## | 0.71 | 0.20 | ######## |
| 725 | *SAC3D1* | 0.65 | 14 | ######## | 0.42 | 0.06 | ######## |
| 726 | *SMC4* | 0.82 | 14 | ######## | 0.56 | 0.11 | ######## |
| 727 | *HMGB3* | 0.65 | 14 | ######## | 0.43 | 0.07 | ######## |
| 728 | *HMGN2* | 1.51 | 14 | ######## | 0.90 | 0.55 | ######## |
| 729 | *TUBA1B* | 1.52 | 14 | ######## | 0.94 | 0.67 | ######## |
| 730 | *HIST1H4C* | 1.31 | 14 | ######## | 0.54 | 0.13 | ######## |
| 731 | *TUBB* | 1.57 | 14 | ######## | 0.87 | 0.49 | ######## |
| 732 | *PCNA* | 0.98 | 14 | ######## | 0.50 | 0.13 | ######## |
| 733 | *NUDT1* | 0.64 | 14 | ######## | 0.53 | 0.14 | ######## |
| 734 | *IDH2* | 0.92 | 14 | ######## | 0.65 | 0.21 | ######## |
| 735 | *SNRNP25* | 0.72 | 14 | ######## | 0.53 | 0.16 | ######## |
| 736 | *HMGB2* | 1.11 | 14 | ######## | 0.79 | 0.38 | ######## |
| 737 | *HMGB1* | 0.95 | 14 | ######## | 0.89 | 0.71 | ######## |
| 738 | *CKS2* | 0.71 | 14 | ######## | 0.61 | 0.22 | ######## |
| 739 | *H2AFV* | 0.89 | 14 | ######## | 0.78 | 0.43 | ######## |
| 740 | *DUT* | 0.87 | 14 | ######## | 0.71 | 0.35 | 3.20E-99 |
| 741 | *NUCKS1* | 0.77 | 14 | ######## | 0.75 | 0.38 | 2.65E-96 |
| 742 | *RPA3* | 0.64 | 14 | 5.59E-99 | 0.59 | 0.23 | 1.88E-94 |
| 743 | *DEK* | 0.81 | 14 | 1.97E-94 | 0.73 | 0.40 | 6.64E-90 |
| 744 | *ANP32B* | 0.84 | 14 | 2.07E-94 | 0.76 | 0.45 | 6.97E-90 |
| 745 | *LSM4* | 0.70 | 14 | 5.21E-90 | 0.72 | 0.38 | 1.76E-85 |
| 746 | *RANBP1* | 0.78 | 14 | 9.57E-83 | 0.73 | 0.41 | 3.23E-78 |

| | | | | | | | |
|---|---|---|---|---|---|---|---|
| 747 | *SLC25A5* | 0.84 | 14 | 2.89E-76 | 0.87 | 0.62 | 9.73E-72 |
| 748 | *RAN* | 0.65 | 14 | 1.75E-64 | 0.81 | 0.56 | 5.89E-60 |
| 749 | *APOE* | 0.82 | 14 | 1.47E-20 | 0.65 | 0.45 | 4.95E-16 |
| 750 | *S100A8* | 0.91 | 14 | 0.003695 | 0.43 | 0.32 | 1 |
| 751 | *SCGB3A1* | 5.51 | 15 | 0 | 0.84 | 0.22 | 0 |
| 752 | *BPIFB1* | 4.11 | 15 | 0 | 0.61 | 0.02 | 0 |
| 753 | *SLPI* | 3.44 | 15 | 0 | 0.93 | 0.18 | 0 |
| 754 | *MSMB* | 3.36 | 15 | 0 | 0.36 | 0.01 | 0 |
| 755 | *WFDC2* | 3.26 | 15 | 0 | 0.89 | 0.08 | 0 |
| 756 | *TFF3* | 2.86 | 15 | 0 | 0.53 | 0.04 | 0 |
| 757 | *LCN2* | 2.74 | 15 | 0 | 0.61 | 0.03 | 0 |
| 758 | *PIGR* | 2.32 | 15 | 0 | 0.68 | 0.05 | 0 |
| 759 | *CXCL17* | 2.11 | 15 | 0 | 0.77 | 0.06 | 0 |
| 760 | *AGR2* | 2.06 | 15 | 0 | 0.67 | 0.06 | 0 |
| 761 | *KRT19* | 1.88 | 15 | 0 | 0.69 | 0.08 | 0 |
| 762 | *TACSTD2* | 1.66 | 15 | 0 | 0.66 | 0.07 | 0 |
| 763 | *KRT18* | 1.60 | 15 | 0 | 0.73 | 0.12 | 0 |
| 764 | *ELF3* | 1.56 | 15 | 0 | 0.65 | 0.07 | 0 |
| 765 | *KLK11* | 1.52 | 15 | 0 | 0.61 | 0.01 | 0 |
| 766 | *CLDN4* | 1.36 | 15 | 0 | 0.60 | 0.06 | 0 |
| 767 | *KRT8* | 1.34 | 15 | 0 | 0.62 | 0.10 | 0 |
| 768 | *TMEM45A* | 1.30 | 15 | 0 | 0.39 | 0.02 | 0 |
| 769 | *TSPAN8* | 1.23 | 15 | 0 | 0.45 | 0.02 | 0 |
| 770 | *FXYD3* | 1.23 | 15 | 0 | 0.63 | 0.07 | 0 |
| 771 | *GSTA1* | 1.21 | 15 | 0 | 0.43 | 0.02 | 0 |
| 772 | *GPRC5A* | 1.20 | 15 | 0 | 0.57 | 0.07 | 0 |
| 773 | *FOLR1* | 1.19 | 15 | 0 | 0.57 | 0.08 | 0 |
| 774 | *AQP5* | 1.17 | 15 | 0 | 0.36 | 0.00 | 0 |
| 775 | *AGR3* | 1.13 | 15 | 0 | 0.49 | 0.06 | 0 |
| 776 | *KRT7* | 1.11 | 15 | 0 | 0.62 | 0.07 | 0 |
| 777 | *TSPAN1* | 1.10 | 15 | 0 | 0.47 | 0.03 | 0 |
| 778 | *AKR1C1* | 1.07 | 15 | 0 | 0.44 | 0.05 | 0 |
| 779 | *S100A14* | 1.07 | 15 | 0 | 0.54 | 0.06 | 0 |
| 780 | *C16orf89* | 1.07 | 15 | 0 | 0.47 | 0.05 | 0 |
| 781 | *CP* | 1.02 | 15 | 0 | 0.44 | 0.01 | 0 |
| 782 | *CXCL6* | 0.98 | 15 | 0 | 0.25 | 0.00 | 0 |
| 783 | *MUC1* | 0.95 | 15 | 0 | 0.57 | 0.07 | 0 |
| 784 | *EPCAM* | 0.88 | 15 | 0 | 0.51 | 0.06 | 0 |
| 785 | *CYP4B1* | 0.87 | 15 | 0 | 0.47 | 0.05 | 0 |
| 786 | *S100P* | 1.73 | 15 | ######## | 0.51 | 0.08 | ######## |
| 787 | *CXCL1* | 2.19 | 15 | ######## | 0.58 | 0.10 | ######## |
| 788 | *SCGB1A1* | 5.91 | 15 | ######## | 0.85 | 0.35 | ######## |
| 789 | *SCGB3A2* | 4.75 | 15 | ######## | 0.51 | 0.09 | ######## |
| 790 | *PERP* | 0.93 | 15 | ######## | 0.51 | 0.09 | ######## |

| | | | | | | | |
|---|---|---|---|---|---|---|---|
| 791 | *MDK* | 1.06 | 15 | ######## | 0.49 | 0.09 | ######## |
| 792 | *SFTPB* | 1.56 | 15 | ######## | 0.59 | 0.15 | ######## |
| 793 | *CYB5A* | 1.47 | 15 | ######## | 0.85 | 0.46 | ######## |
| 794 | *SOX4* | 1.16 | 15 | ######## | 0.51 | 0.13 | ######## |
| 795 | *GDF15* | 1.05 | 15 | ######## | 0.36 | 0.07 | ######## |
| 796 | *NCOA7* | 1.05 | 15 | ######## | 0.52 | 0.16 | ######## |
| 797 | *MGST1* | 1.05 | 15 | ######## | 0.74 | 0.34 | ######## |
| 798 | *MGP* | 1.06 | 15 | ######## | 0.51 | 0.16 | 1.82E-96 |
| 799 | *SPINT2* | 0.97 | 15 | ######## | 0.66 | 0.31 | 1.16E-95 |
| 800 | *CXCL8* | 1.48 | 15 | 7.42E-78 | 0.63 | 0.33 | 2.50E-73 |
| 801 | *CCL21* | 5.30 | 16 | 0 | 0.97 | 0.03 | 0 |
| 802 | *TFF3* | 3.05 | 16 | 0 | 0.93 | 0.03 | 0 |
| 803 | *MMRN1* | 2.49 | 16 | 0 | 0.83 | 0.02 | 0 |
| 804 | *GNG11* | 2.44 | 16 | 0 | 0.94 | 0.13 | 0 |
| 805 | *IGFBP7* | 2.43 | 16 | 0 | 0.96 | 0.26 | 0 |
| 806 | *RGS16* | 2.36 | 16 | 0 | 0.69 | 0.07 | 0 |
| 807 | *ADIRF* | 2.17 | 16 | 0 | 0.90 | 0.16 | 0 |
| 808 | *SNCG* | 2.06 | 16 | 0 | 0.77 | 0.03 | 0 |
| 809 | *SDPR* | 2.06 | 16 | 0 | 0.87 | 0.09 | 0 |
| 810 | *TFPI* | 2.06 | 16 | 0 | 0.89 | 0.16 | 0 |
| 811 | *AKAP12* | 2.00 | 16 | 0 | 0.75 | 0.07 | 0 |
| 812 | *PPFIBP1* | 1.94 | 16 | 0 | 0.81 | 0.05 | 0 |
| 813 | *IGFBP5* | 1.90 | 16 | 0 | 0.40 | 0.04 | 0 |
| 814 | *ECSCR.1* | 1.83 | 16 | 0 | 0.81 | 0.12 | 0 |
| 815 | *CLDN5* | 1.79 | 16 | 0 | 0.88 | 0.10 | 0 |
| 816 | *NNMT* | 1.77 | 16 | 0 | 0.87 | 0.17 | 0 |
| 817 | *APP* | 1.71 | 16 | 0 | 0.86 | 0.22 | 0 |
| 818 | *LYVE1* | 1.56 | 16 | 0 | 0.50 | 0.01 | 0 |
| 819 | *CLU* | 1.55 | 16 | 0 | 0.81 | 0.14 | 0 |
| 820 | *HYAL2* | 1.53 | 16 | 0 | 0.76 | 0.13 | 0 |
| 821 | *PDPN* | 1.50 | 16 | 0 | 0.61 | 0.01 | 0 |
| 822 | *CNN3* | 1.47 | 16 | 0 | 0.81 | 0.14 | 0 |
| 823 | *APOLD1* | 1.39 | 16 | 0 | 0.57 | 0.04 | 0 |
| 824 | *TM4SF1* | 1.37 | 16 | 0 | 0.84 | 0.17 | 0 |
| 825 | *TIMP3* | 1.36 | 16 | 0 | 0.84 | 0.14 | 0 |
| 826 | *FXYD6* | 1.35 | 16 | 0 | 0.65 | 0.08 | 0 |
| 827 | *TM4SF18* | 1.34 | 16 | 0 | 0.54 | 0.02 | 0 |
| 828 | *CRIP2* | 1.28 | 16 | 0 | 0.75 | 0.12 | 0 |
| 829 | *KANK3* | 1.27 | 16 | 0 | 0.63 | 0.03 | 0 |
| 830 | *NTS* | 1.27 | 16 | 0 | 0.32 | 0.00 | 0 |
| 831 | *RAMP2* | 1.26 | 16 | 0 | 0.70 | 0.07 | 0 |
| 832 | *SLC9A3R2* | 1.24 | 16 | 0 | 0.66 | 0.10 | 0 |
| 833 | *CAV1* | 1.20 | 16 | 0 | 0.80 | 0.15 | 0 |
| 834 | *IGFBP4* | 1.18 | 16 | 0 | 0.69 | 0.11 | 0 |

| | | | | | | | |
|---|---|---|---|---|---|---|---|
| 835 | *FSCN1* | 1.18 | 16 | 0 | 0.54 | 0.03 | 0 |
| 836 | *GGT5* | 1.16 | 16 | 0 | 0.51 | 0.02 | 0 |
| 837 | *IGF1* | 1.16 | 16 | 0 | 0.41 | 0.03 | 0 |
| 838 | *C16orf62* | 1.11 | 16 | 0 | 0.55 | 0.07 | 0 |
| 839 | *PROCR* | 1.30 | 16 | ######## | 0.60 | 0.10 | ######## |
| 840 | *PLPP1* | 1.17 | 16 | ######## | 0.55 | 0.08 | ######## |
| 841 | *MYC* | 1.33 | 16 | ######## | 0.64 | 0.13 | ######## |
| 842 | *EFEMP1* | 1.25 | 16 | ######## | 0.43 | 0.06 | ######## |
| 843 | *HES1* | 1.51 | 16 | ######## | 0.63 | 0.15 | ######## |
| 844 | *RAB11A* | 1.27 | 16 | ######## | 0.82 | 0.36 | ######## |
| 845 | *HSPB1* | 1.26 | 16 | ######## | 0.94 | 0.55 | ######## |
| 846 | *CD59* | 1.31 | 16 | ######## | 0.90 | 0.48 | ######## |
| 847 | *IFITM3* | 1.17 | 16 | ######## | 0.92 | 0.49 | ######## |
| 848 | *RHOC* | 1.09 | 16 | ######## | 0.74 | 0.27 | ######## |
| 849 | *GYPC* | 1.21 | 16 | ######## | 0.82 | 0.41 | ######## |
| 850 | *HSPA1A* | 1.23 | 16 | 7.41E-53 | 0.62 | 0.36 | 2.50E-48 |
| 851 | *IGKC* | 5.93 | 17 | 0 | 0.64 | 0.08 | 0 |
| 852 | *IGHM* | 4.45 | 17 | 0 | 0.42 | 0.01 | 0 |
| 853 | *JCHAIN* | 4.03 | 17 | 0 | 0.37 | 0.02 | 0 |
| 854 | *MZB1* | 2.13 | 17 | 0 | 0.30 | 0.01 | 0 |
| 855 | *CD79A* | 1.99 | 17 | 0 | 0.60 | 0.00 | 0 |
| 856 | *MS4A1* | 1.58 | 17 | 0 | 0.41 | 0.00 | 0 |
| 857 | *LY9* | 1.13 | 17 | 0 | 0.31 | 0.01 | 0 |
| 858 | *TNFRSF13C* | 1.00 | 17 | 0 | 0.27 | 0.00 | 0 |
| 859 | *BANK1* | 0.99 | 17 | 0 | 0.27 | 0.01 | 0 |
| 860 | *GNG7* | 0.91 | 17 | 0 | 0.33 | 0.02 | 0 |
| 861 | *CCR7* | 1.23 | 17 | ######## | 0.31 | 0.02 | ######## |
| 862 | *IGLC3* | 4.51 | 17 | ######## | 0.26 | 0.02 | ######## |
| 863 | *IGLC2* | 4.74 | 17 | ######## | 0.33 | 0.03 | ######## |
| 864 | *IGHA1* | 4.76 | 17 | ######## | 0.30 | 0.04 | ######## |
| 865 | *ISG20* | 1.08 | 17 | ######## | 0.64 | 0.23 | 8.60E-96 |
| 866 | *ITM2C* | 0.93 | 17 | 2.27E-98 | 0.26 | 0.04 | 7.65E-94 |
| 867 | *HERPUD1* | 1.45 | 17 | 5.79E-88 | 0.76 | 0.47 | 1.95E-83 |
| 868 | *RPL3* | 0.71 | 17 | 1.06E-87 | 0.99 | 0.94 | 3.56E-83 |
| 869 | *LTB* | 1.14 | 17 | 1.37E-87 | 0.35 | 0.08 | 4.62E-83 |
| 870 | *RPL13A* | 0.67 | 17 | 8.03E-74 | 1.00 | 0.97 | 2.71E-69 |
| 871 | *RPL13* | 0.62 | 17 | 3.87E-67 | 0.99 | 0.97 | 1.30E-62 |
| 872 | *RPL23A* | 0.64 | 17 | 5.21E-65 | 0.98 | 0.93 | 1.76E-60 |
| 873 | *RHOH* | 0.86 | 17 | 4.15E-64 | 0.45 | 0.15 | 1.40E-59 |
| 874 | *RPL18A* | 0.68 | 17 | 9.92E-64 | 0.98 | 0.95 | 3.34E-59 |
| 875 | *CXCR4* | 1.04 | 17 | 1.76E-63 | 0.72 | 0.41 | 5.91E-59 |
| 876 | *RPS8* | 0.66 | 17 | 2.60E-61 | 0.96 | 0.92 | 8.77E-57 |
| 877 | *RPS6* | 0.61 | 17 | 3.57E-60 | 0.99 | 0.95 | 1.20E-55 |
| 878 | *RPS5* | 0.62 | 17 | 2.81E-59 | 0.94 | 0.89 | 9.48E-55 |

| | | | | | | | |
|---|---|---|---|---|---|---|---|
| 879 | *RPL21* | 0.66 | 17 | 4.29E-59 | 0.99 | 0.96 | 1.45E-54 |
| 880 | *EEF1B2* | 0.75 | 17 | 1.74E-56 | 0.89 | 0.75 | 5.87E-52 |
| 881 | *RPL4* | 0.62 | 17 | 4.37E-49 | 0.92 | 0.81 | 1.47E-44 |
| 882 | *RPS27* | 0.66 | 17 | 4.94E-49 | 0.97 | 0.94 | 1.67E-44 |
| 883 | *RPS23* | 0.61 | 17 | 2.45E-48 | 0.97 | 0.93 | 8.26E-44 |
| 884 | *CD37* | 1.04 | 17 | 1.61E-46 | 0.64 | 0.42 | 5.43E-42 |
| 885 | *BIRC3* | 0.84 | 17 | 5.50E-44 | 0.53 | 0.25 | 1.85E-39 |
| 886 | *EEF2* | 0.66 | 17 | 1.01E-38 | 0.82 | 0.71 | 3.41E-34 |
| 887 | *TSC22D3* | 0.67 | 17 | 1.75E-38 | 0.77 | 0.61 | 5.90E-34 |
| 888 | *GLTSCR2* | 0.63 | 17 | 2.38E-36 | 0.77 | 0.61 | 8.01E-32 |
| 889 | *EZR* | 0.86 | 17 | 5.21E-34 | 0.59 | 0.39 | 1.75E-29 |
| 890 | *CD83* | 1.18 | 17 | 4.86E-25 | 0.45 | 0.30 | 1.64E-20 |
| 891 | *GPR183* | 0.75 | 17 | 1.13E-21 | 0.39 | 0.21 | 3.80E-17 |
| 892 | *FKBP11* | 0.76 | 17 | 2.35E-21 | 0.27 | 0.13 | 7.91E-17 |
| 893 | *BTG1* | 0.72 | 17 | 8.01E-20 | 0.79 | 0.79 | 2.70E-15 |
| 894 | *SMAP2* | 0.62 | 17 | 8.48E-19 | 0.37 | 0.22 | 2.86E-14 |
| 895 | *SMCHD1* | 0.62 | 17 | 1.06E-18 | 0.33 | 0.18 | 3.57E-14 |
| 896 | *YPEL5* | 0.75 | 17 | 8.88E-18 | 0.49 | 0.36 | 2.99E-13 |
| 897 | *ZNF331* | 0.73 | 17 | 2.84E-17 | 0.47 | 0.33 | 9.58E-13 |
| 898 | *RPL17* | 0.64 | 17 | 1.99E-14 | 0.61 | 0.53 | 6.70E-10 |
| 899 | *IRF8* | 0.83 | 17 | 4.67E-09 | 0.30 | 0.23 | 0.000157 |
| 900 | *SSR4* | 0.85 | 17 | 0.001465 | 0.52 | 0.62 | 1 |
| 901 | *HBD* | 3.63 | 18 | 0 | 1.00 | 0.00 | 0 |
| 902 | *ALAS2* | 3.03 | 18 | 0 | 1.00 | 0.00 | 0 |
| 903 | *CA1* | 3.01 | 18 | 0 | 1.00 | 0.00 | 0 |
| 904 | *AHSP* | 2.77 | 18 | 0 | 1.00 | 0.00 | 0 |
| 905 | *HBM* | 2.36 | 18 | 0 | 0.77 | 0.00 | 0 |
| 906 | *SLC4A1* | 1.56 | 18 | 0 | 0.85 | 0.00 | 0 |
| 907 | *KRT1* | 1.23 | 18 | 0 | 0.69 | 0.00 | 0 |
| 908 | *HEMGN* | 0.85 | 18 | 0 | 0.46 | 0.00 | 0 |
| 909 | *HBQ1* | 0.84 | 18 | 0 | 0.46 | 0.00 | 0 |
| 910 | *GYPA* | 0.70 | 18 | 0 | 0.31 | 0.00 | 0 |
| 911 | *GYPB* | 0.69 | 18 | 0 | 0.31 | 0.00 | 0 |
| 912 | *HBA1* | 7.01 | 18 | ######## | 1.00 | 0.02 | ######## |
| 913 | *EPB42* | 0.78 | 18 | 1.40E-82 | 0.46 | 0.01 | 4.71E-78 |
| 914 | *TRIM58* | 0.87 | 18 | 6.60E-77 | 0.39 | 0.01 | 2.22E-72 |
| 915 | *HBA2* | 7.48 | 18 | 4.24E-64 | 1.00 | 0.05 | 1.43E-59 |
| 916 | *SNCA* | 2.29 | 18 | 4.16E-44 | 0.92 | 0.06 | 1.40E-39 |
| 917 | *STRADB* | 1.50 | 18 | 2.53E-42 | 1.00 | 0.07 | 8.51E-38 |
| 918 | *HBB* | 8.48 | 18 | 1.07E-39 | 1.00 | 0.08 | 3.59E-35 |
| 919 | *DMTN* | 0.72 | 18 | 3.81E-35 | 0.39 | 0.01 | 1.28E-30 |
| 920 | *FECH* | 1.35 | 18 | 7.43E-33 | 0.77 | 0.05 | 2.50E-28 |
| 921 | *BPGM* | 1.63 | 18 | 2.38E-31 | 0.85 | 0.07 | 8.02E-27 |
| 922 | *DCAF12* | 1.67 | 18 | 9.24E-28 | 0.92 | 0.10 | 3.11E-23 |

| | | | | | | | |
|---|---|---|---|---|---|---|---|
| 923 | *GMPR* | 1.63 | 18 | 4.24E-25 | 0.77 | 0.07 | 1.43E-20 |
| 924 | *SLC25A37* | 2.10 | 18 | 5.40E-20 | 1.00 | 0.18 | 1.82E-15 |
| 925 | *PDZK1IP1* | 0.66 | 18 | 9.19E-17 | 0.39 | 0.03 | 3.10E-12 |
| 926 | *SLC25A39* | 1.82 | 18 | 4.56E-16 | 1.00 | 0.24 | 1.54E-11 |
| 927 | *MKRN1* | 1.48 | 18 | 2.37E-14 | 1.00 | 0.26 | 7.98E-10 |
| 928 | *EPB41* | 0.79 | 18 | 3.67E-11 | 0.46 | 0.05 | 1.24E-06 |
| 929 | *GLRX5* | 1.26 | 18 | 4.25E-11 | 0.77 | 0.17 | 1.43E-06 |
| 930 | *GYPC* | 1.22 | 18 | 5.45E-09 | 1.00 | 0.42 | 0.000184 |
| 931 | *SELENBP1* | 0.66 | 18 | 6.27E-09 | 0.46 | 0.07 | 0.000211 |
| 932 | *NCOA4* | 1.83 | 18 | 1.26E-08 | 0.77 | 0.25 | 0.000426 |
| 933 | *NUDT4* | 1.01 | 18 | 1.32E-08 | 0.77 | 0.21 | 0.000443 |
| 934 | *CA2* | 0.65 | 18 | 1.32E-08 | 0.62 | 0.12 | 0.000445 |
| 935 | *FAM210B* | 1.16 | 18 | 5.06E-08 | 0.54 | 0.11 | 0.001706 |
| 936 | *FKBP8* | 1.13 | 18 | 7.47E-08 | 0.92 | 0.39 | 0.002517 |
| 937 | *FBXO7* | 1.10 | 18 | 7.56E-08 | 0.77 | 0.25 | 0.002547 |
| 938 | *GSPT1* | 1.07 | 18 | 7.80E-08 | 0.85 | 0.30 | 0.002629 |
| 939 | *ADIPOR1* | 0.96 | 18 | 5.27E-07 | 0.85 | 0.33 | 0.017741 |
| 940 | *UBB* | 1.24 | 18 | 9.70E-07 | 1.00 | 0.80 | 0.032698 |
| 941 | *ISCA1* | 1.11 | 18 | 1.76E-06 | 0.69 | 0.22 | 0.059148 |
| 942 | *BNIP3L* | 1.18 | 18 | 3.72E-06 | 0.85 | 0.41 | 0.125346 |
| 943 | *BSG* | 0.94 | 18 | 5.96E-06 | 0.92 | 0.47 | 0.200724 |
| 944 | *RNF10* | 1.05 | 18 | 6.75E-06 | 0.62 | 0.20 | 0.227495 |
| 945 | *SIAH2* | 0.77 | 18 | 6.86E-06 | 0.54 | 0.13 | 0.2311 |
| 946 | *PRDX2* | 0.77 | 18 | 9.92E-06 | 0.69 | 0.22 | 0.334406 |
| 947 | *BLVRB* | 0.99 | 18 | 1.85E-05 | 0.85 | 0.41 | 0.624257 |
| 948 | *C9orf78* | 0.95 | 18 | 3.37E-05 | 0.69 | 0.25 | 1 |
| 949 | *BCL2L1* | 0.65 | 18 | 4.49E-05 | 0.54 | 0.17 | 1 |
| 950 | *PIP4K2A* | 0.71 | 18 | 5.73E-05 | 0.54 | 0.16 | 1 |

**Supplementary Table 2. Top upregulated Gene Ontology pathways in pericytes/SMCs in IPAH**

| **Pathways in IPAH pericytes** | **# of genes** | **FE** | **FDR** |
|---|---|---|---|
| Negative regulation of cell development | 20 | 6.26 | 3.08E-06 |
| Anatomical structure morphogenesis | 51 | 2.5 | 8.97E-06 |
| Negative regulation of developmental process | 31 | 3.42 | 1.88E-05 |
| Negative regulation of cell differentiation | 26 | 3.83 | 2.56E-05 |
| Extracellular matrix organization | 18 | 5.53 | 2.70E-05 |
| Extracellular structure organization | 19 | 5.13 | 2.79E-05 |
| Circulatory system development | 29 | 3.5 | 2.85E-05 |
| Negative regulation of multicellular organismal process | 34 | 2.97 | 3.21E-05 |
| System development | 78 | 1.84 | 3.46E-05 |

| | | | |
|---|---|---|---|
| Regulation of intracellular signal transduction | 44 | 2.5 | 3.68E-05 |
| Animal organ morphogenesis | 29 | 3.21 | 5.60E-05 |
| Regulation of cell differentiation | 43 | 2.47 | 5.83E-05 |
| Negative regulation of neurogenesis | 16 | 5.76 | 5.90E-05 |
| Cardiovascular system development | 21 | 4.16 | 6.84E-05 |
| Animal organ development | 61 | 2 | 7.11E-05 |
| Tube development | 27 | 3.32 | 7.18E-05 |
| Multicellular organism development | 83 | 1.72 | 7.69E-05 |
| Negative regulation of nervous system development | 16 | 5.42 | 8.01E-05 |
| Blood vessel development | 20 | 4.24 | 8.28E-05 |
| Regulation of MAPK cascade | 25 | 3.47 | 8.71E-05 |
| Cellular process | 173 | 1.23 | 1.30E-04 |
| Regulation of signal transduction | 62 | 1.95 | 1.34E-04 |
| Regulation of cell communication | 66 | 1.87 | 1.35E-04 |
| Vasculature development | 20 | 4.04 | 1.41E-04 |
| Regulation of cell migration | 26 | 3.24 | 1.45E-04 |
| Regulation of developmental process | 52 | 2.1 | 1.47E-04 |
| Anatomical structure development | 86 | 1.66 | 1.56E-04 |
| Biological regulation | 155 | 1.3 | 1.68E-04 |
| Regulation of signaling | 66 | 1.85 | 2.17E-04 |
| Eye morphogenesis | 11 | 7.6 | 2.34E-04 |
| Developmental process | 89 | 1.61 | 2.35E-04 |
| Eye development | 16 | 4.75 | 2.39E-04 |
| Regulation of cell population proliferation | 38 | 2.42 | 2.42E-04 |
| Tube morphogenesis | 22 | 3.51 | 2.46E-04 |
| Visual system development | 16 | 4.69 | 2.61E-04 |
| Sensory system development | 16 | 4.63 | 3.04E-04 |
| Blood vessel morphogenesis | 17 | 4.33 | 3.15E-04 |
| Regulation of cell motility | 26 | 3.01 | 3.25E-04 |
| Heart development | 19 | 3.83 | 3.82E-04 |
| Regulation of multicellular organismal process | 57 | 1.9 | 5.35E-04 |
| Regulation of cell development | 26 | 2.88 | 6.22E-04 |
| Regulation of MAP kinase activity | 15 | 4.61 | 6.28E-04 |
| Biological process | 195 | 1.13 | 6.52E-04 |
| Sensory organ development | 19 | 3.65 | 6.72E-04 |
| Ossification | 13 | 5.27 | 7.01E-04 |
| Sensory organ morphogenesis | 13 | 5.23 | 7.45E-04 |
| Regulation of response to stimulus | 72 | 1.69 | 7.93E-04 |
| Negative regulation of cellular process | 75 | 1.66 | 8.22E-04 |
| Cell differentiation | 63 | 1.78 | 8.36E-04 |
| Regulation of locomotion | 26 | 2.78 | 9.77E-04 |

| | | | |
|---|---|---|---|
| Cellular developmental process | 64 | 1.76 | 9.77E-04 |
| Regulation of protein serine/threonine kinase activity | 18 | 3.68 | 9.92E-04 |
| Negative regulation of neuron differentiation | 12 | 5.51 | 1.01E-03 |
| Regulation of cellular component movement | 26 | 2.76 | 1.07E-03 |
| Regulation of biological process | 145 | 1.28 | 1.45E-03 |
| Regulation of anatomical structure morphogenesis | 27 | 2.62 | 1.68E-03 |
| Positive regulation of protein kinase activity | 18 | 3.5 | 1.70E-03 |
| Anatomical structure formation involved in morphogenesis | 24 | 2.81 | 1.83E-03 |
| Cell development | 35 | 2.25 | 2.05E-03 |
| Positive regulation of cell migration | 17 | 3.59 | 2.14E-03 |
| Multicellular organismal process | 98 | 1.46 | 2.16E-03 |
| Regulation of phospholipase C activity | 6 | 13.72 | 2.32E-03 |
| Cellular response to growth factor stimulus | 17 | 3.53 | 2.50E-03 |
| Regulation of protein kinase activity | 22 | 2.9 | 2.63E-03 |
| Supramolecular fiber organization | 16 | 3.69 | 2.65E-03 |
| Camera-type eye development | 13 | 4.45 | 2.79E-03 |
| Positive regulation of MAP kinase activity | 12 | 4.79 | 2.89E-03 |
| Positive regulation of biological process | 89 | 1.5 | 2.90E-03 |
| Positive regulation of protein modification process | 29 | 2.46 | 2.92E-03 |
| Positive regulation of cell motility | 17 | 3.44 | 3.15E-03 |
| Negative regulation of biological process | 79 | 1.56 | 3.15E-03 |
| Nervous system development | 44 | 1.95 | 3.50E-03 |
| Positive regulation of protein phosphorylation | 25 | 2.59 | 3.70E-03 |
| Positive regulation of kinase activity | 18 | 3.22 | 3.89E-03 |
| Positive regulation of cellular component movement | 17 | 3.35 | 4.07E-03 |
| Response to growth factor | 17 | 3.33 | 4.41E-03 |
| Locomotion | 29 | 2.33 | 5.08E-03 |
| Positive regulation of MAPK cascade | 17 | 3.26 | 5.42E-03 |

**Supplementary Table 3. Top differentially upregulated genes in IPAH fibroblasts (10% FDR)**

| **10% FDR genes** | **Fold change** | **10% FDR genes** | **Fold change** |
|---|---|---|---|
| *NUDT3* | 4.45 | *SFRP2* | 4.70 |
| *APCDD1* | 3.87 | *PSD3* | 4.00 |
| *FTX* | 3.81 | *C20orf27* | 3.30 |
| *MMD* | 3.47 | *KIAA0907* | 2.68 |
| *RBM26* | 3.34 | *SLC16A7* | 2.58 |

| | | | |
|---|---|---|---|
| *SLC35C2* | 3.28 | *UBE2G2* | 2.55 |
| *IL11RA* | 3.21 | *API5* | 2.45 |
| *GAMT* | 3.20 | *NAPA* | 2.44 |
| *BEX4* | 3.13 | *TNPO1* | 2.28 |
| *CHID1* | 2.54 | *CTSF* | 2.22 |
| *COL8A1* | 2.49 | *GPBP1L1* | 2.20 |
| *CHPF* | 2.34 | *ZCCHC24* | 2.18 |
| *FAM228B* | 2.30 | *MXD4* | 2.13 |
| *ROCK1* | 2.29 | *CASS4* | 2.10 |
| *SFTPC* | 2.19 | *ETV1* | 2.08 |
| *ZFP36L1* | 2.16 | *RNASET2* | 2.06 |
| *RUNX1T1* | 2.07 | *FKBP8* | 2.04 |
| *ITGA8* | 2.06 | *NBL1* | 2.03 |
| *SRSF7* | 2.05 | *PTGER4* | 2.02 |
| *IFT43* | 1.95 | *TMEM98* | 1.94 |
| *WDR91* | 1.94 | *FAM229B* | 1.90 |
| *PTGER1* | 1.94 | *RTF1* | 1.89 |
| *DHRS4L2* | 1.91 | *CTSS* | 1.74 |
| *SS18* | 1.85 | *DDB1* | 1.67 |
| *PNN* | 1.80 | *SEC63* | 1.62 |
| *BRD4* | 1.75 | *MEP1B* | 1.56 |
| *RING1* | 1.73 | *SMPD1* | 1.55 |
| *MAGEH1* | 1.69 | *FDX1* | 1.54 |
| *C4orf3* | 1.67 | *MTPN* | 1.53 |
| *SLC2A3* | 1.53 | *WDR33* | 1.52 |
| *FKBP3* | 1.50 | | |

**Supplementary Table 4. Top differentially upregulated Gene Ontology pathways in fibroblasts in IPAH (4A) and genes associated with select Gene Ontology pathways in IPAH fibroblasts (4B)**

| 4A. Pathways in IPAH fibroblasts | # of genes | FE | FDR |
|---|---|---|---|
| Extracellular matrix organization | 10 | 8.07 | 8.67E-03 |
| Cell adhesion | 15 | 4.41 | 1.06E-02 |
| Biological adhesion | 15 | 4.28 | 7.67E-03 |
| Extracellular structure organization | 10 | 7.12 | 6.51E-03 |
| Regulation of cellular component movement | 15 | 4.13 | 9.57E-03 |
| Regulation of cell motility | 14 | 4.21 | 1.44E-02 |
| Regulation of cell migration | 13 | 4.19 | 2.96E-02 |
| Regulation of locomotion | 14 | 3.89 | 2.59E-02 |
| Convergent extension | 3 | 58.43 | 5.43E-02 |
| Response to transforming growth factor beta | 6 | 10.23 | 5.18E-02 |
| Regulation of Wnt signaling pathway, planar cell polarity pathway | 3 | 54.54 | 5.32E-02 |
| Regulation of establishment of planar polarity involved in neural tube closure | 3 | 54.54 | 4.88E-02 |

| 4B. Extracellular matrix organization pathway | Fold change | Regulation of WNT signaling pathway | Fold change | Response to TGF beta pathway | Fold change |
|---|---|---|---|---|---|
| *SFRP2* | 4.70 | *NPHP3* | 5.40 | *MXRA5* | 3.86 |
| *MFAP4* | 2.53 | *SFRP2* | 4.70 | *ZYX* | 3.08 |
| *A2M* | 2.45 | *GPC3* | 1.95 | *ZFP36L1* | 2.16 |
| *DPT* | 2.36 | | | *LTBP3* | 2.13 |
| *SH3PXD2A* | 2.33 | | | *ADAM9* | 1.77 |
| *ECM2* | 2.23 | | | | |
| *COL16A1* | 2.16 | | | | |
| *ITGA8* | 2.06 | | | | |
| *CTSO* | 1.87 | | | | |
| *MMP2* | 1.78 | | | | |

**Supplementary Table 5. Top differentially upregulated genes in SPP1 macrophages and monocytes (10% FDR)**

| SPP1 macrophages | | Monocytes | |
|---|---|---|---|
| **Genes** | **Fold change** | **Genes** | **Fold change** |
| *CCDC180* | 4.29 | *CAMKK2* | 2.29 |
| *AVPI1* | 1.59 | *MPPE1* | 2.12 |
| *DPEP2* | 2.28 | *PRPF8* | 2.23 |
| *TMEM134* | 1.73 | *PDGFB* | 3.41 |
| *DZIP1L* | 2.18 | *ARL6IP4* | 1.50 |
| *ZDHHC12* | 1.55 | *MTRR* | 2.26 |
| *ALDH2* | 2.60 | *LKAAEAR1* | 23.0 |
| *GALNS* | 2.02 | *EEF2* | 1.50 |
| *HOXB-AS1* | 3.27 | *PROK2* | 3.64 |
| *C9orf16* | 1.60 | *FXYD5* | 1.74 |
| *SPI1* | 1.64 | *SFTPC* | 2.50 |
| *DNAL1* | 1.60 | *PKN1* | 2.32 |
| *CRACR2B* | 3.37 | *TACC3* | 3.46 |
| *HLA-DQA1* | 5.05 | *GLB1L* | 3.56 |
| *C20orf27* | 1.75 | *C10orf11* | 2.05 |
| *FCGR1A* | 4.90 | *GLG1* | 2.12 |
| *APOC1* | 1.78 | | |
| *IFT22* | 1.85 | | |

**Supplementary Table 6. Most significantly upregulated genes in IPAH by bulk microarray**

| Gene | P value | Fold change |
|---|---|---|
| *HBB* | 3.54E-09 | 5.99 |
| *FXD7* | 2.66E-08 | 1.78 |
| *HBA2///HBA3* | 4.15E-08 | 4.46 |
| *LTBP1* | 4.15E-08 | 2.00 |
| *GGTA1P* | 2.12E-07 | 1.74 |
| *PDE7B* | 2.49E-07 | 1.88 |
| *ECM2* | 1.21E-06 | 1.81 |
| *RARRES2* | 1.21E-06 | 1.67 |
| *ETV5* | 1.27E-06 | 1.90 |
| *PDE3A* | 1.31E-06 | 2.19 |
