# Supplementary material for: Transcriptional profiling of lung cell populations in idiopathic pulmonary arterial hypertension
Source: Pulm Circ. 2020 Feb 28;10(1):???. doi: 10.1177/2045894020908782 (PMC7052475; doi:10.1177/2045894020908782)
Supplement: PUL908782 Supplemental Material - Supplemental material for Transcriptional profiling of lung cell populations in idiopathic pulmonary arterial hypertension [file PUL908782_Supplemental_Material.pptx]

## Slide 1
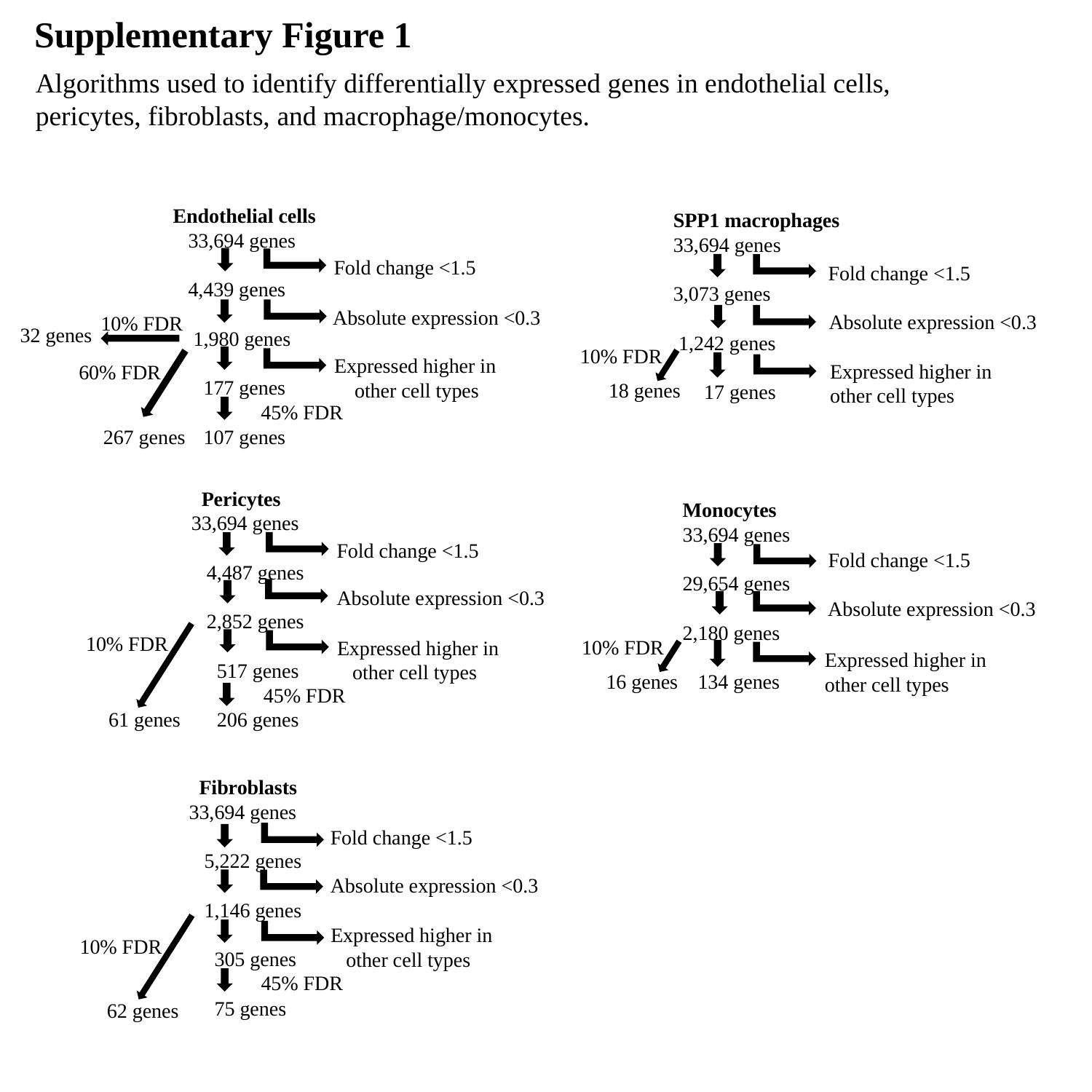

Supplementary Figure 1
Algorithms used to identify differentially expressed genes in endothelial cells, pericytes, fibroblasts, and macrophage/monocytes.
Endothelial cells
 33,694 genes
 4,439 genes
 1,980 genes
 177 genes
 107 genes
SPP1 macrophages
33,694 genes
3,073 genes
 1,242 genes
 17 genes
Fold change <1.5
Fold change <1.5
Absolute expression <0.3
Absolute expression <0.3
10% FDR
32 genes
10% FDR
Expressed higher in
 other cell types
Expressed higher in
other cell types
60% FDR
18 genes
45% FDR
 267 genes
 Pericytes
33,694 genes
 4,487 genes
 2,852 genes
 517 genes
 206 genes
Monocytes
33,694 genes
29,654 genes
2,180 genes
 134 genes
Fold change <1.5
Fold change <1.5
Absolute expression <0.3
Absolute expression <0.3
10% FDR
10% FDR
Expressed higher in
 other cell types
Expressed higher in
other cell types
16 genes
45% FDR
61 genes
 Fibroblasts
33,694 genes
 5,222 genes
 1,146 genes
 305 genes
 75 genes
Fold change <1.5
Absolute expression <0.3
Expressed higher in
 other cell types
10% FDR
45% FDR
62 genes

## Slide 2
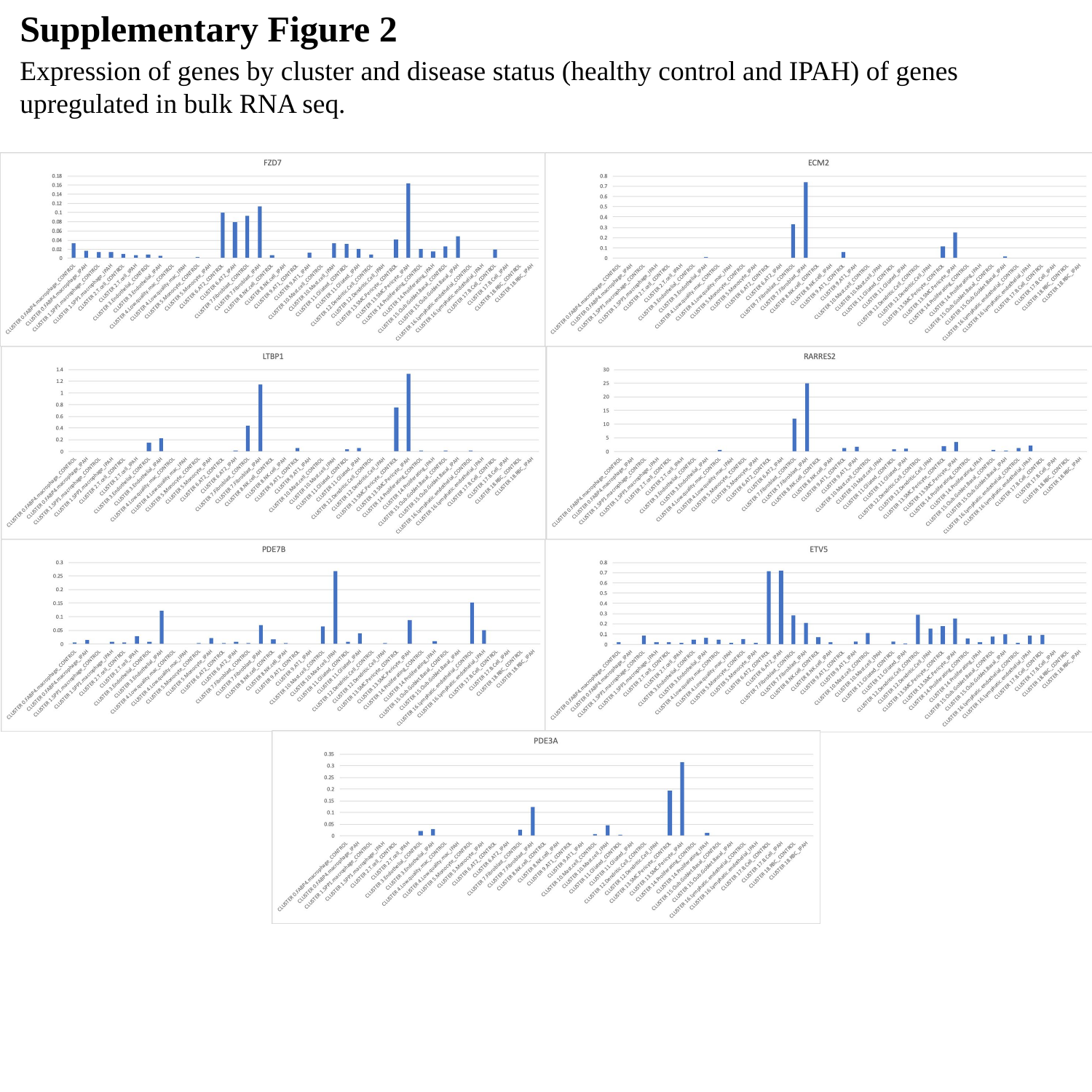

Supplementary Figure 2
Expression of genes by cluster and disease status (healthy control and IPAH) of genes upregulated in bulk RNA seq.

## Slide 3
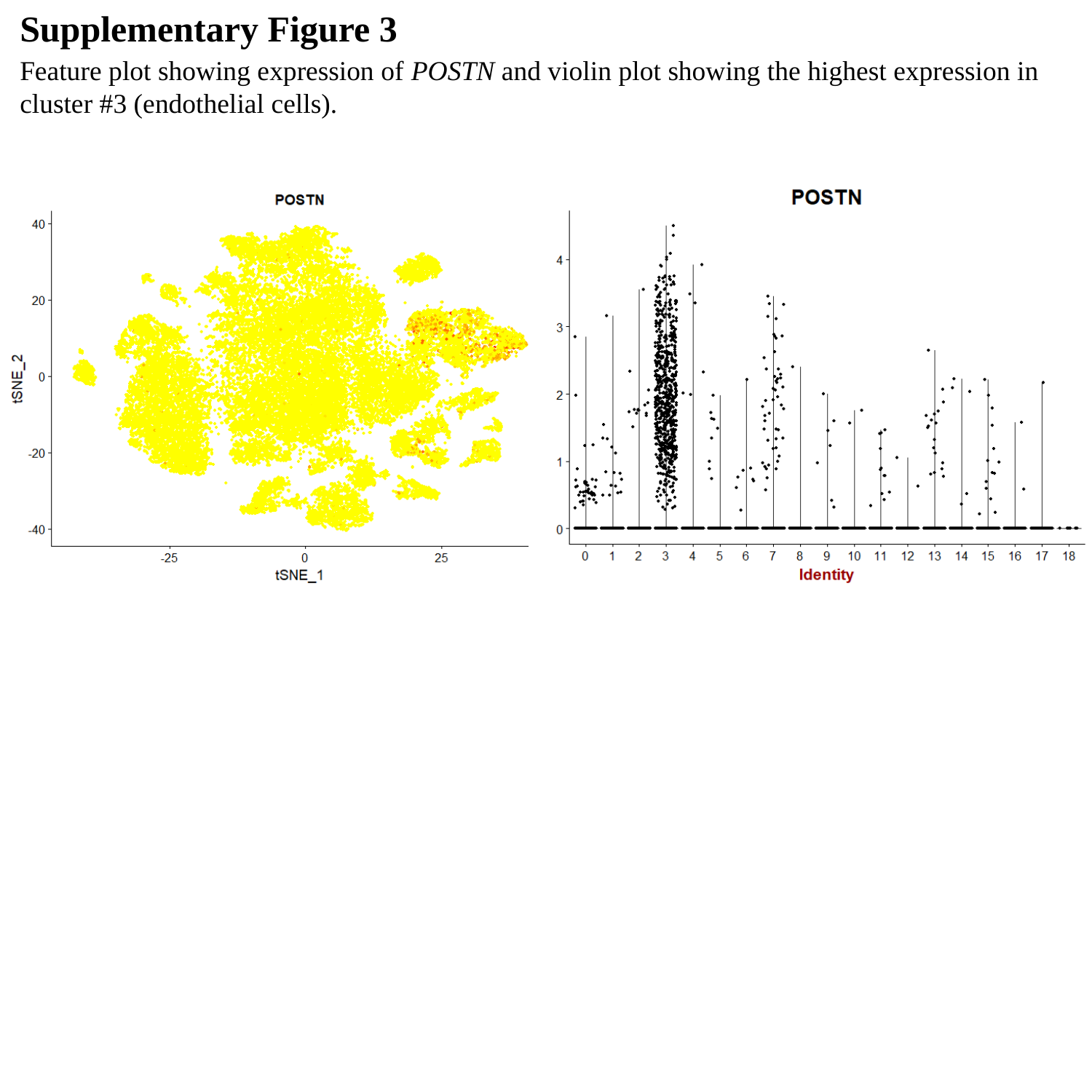

Supplementary Figure 3
Feature plot showing expression of POSTN and violin plot showing the highest expression in cluster #3 (endothelial cells).
